# Synergetic catalysis via zeolite-encapsulated PdCu alloy for highly efficient CO_2_ hydrogenation to ethanol

**DOI:** 10.1093/nsr/nwag303

**Published:** 2026-05-23

**Authors:** Di Xu, Shaojia Song, Guoqiang Hou, Ruosong He, Haifeng Fan, Yangyang Li, Siyi Huang, Hanzhu Zhang, Baojun Wang, Riguang Zhang, Mingyue Ding

**Affiliations:** School of Power and Mechanical Engineering, Wuhan University, Wuhan 430072, China; State Key Laboratory of Clean and Efficient Coal Utilization, College of Chemistry and Chemical Engineering, Taiyuan University of Technology, Taiyuan 030024, China; School of Power and Mechanical Engineering, Wuhan University, Wuhan 430072, China; School of Power and Mechanical Engineering, Wuhan University, Wuhan 430072, China; School of Power and Mechanical Engineering, Wuhan University, Wuhan 430072, China; School of Power and Mechanical Engineering, Wuhan University, Wuhan 430072, China; School of Power and Mechanical Engineering, Wuhan University, Wuhan 430072, China; School of Power and Mechanical Engineering, Wuhan University, Wuhan 430072, China; State Key Laboratory of Clean and Efficient Coal Utilization, College of Chemistry and Chemical Engineering, Taiyuan University of Technology, Taiyuan 030024, China; State Key Laboratory of Clean and Efficient Coal Utilization, College of Chemistry and Chemical Engineering, Taiyuan University of Technology, Taiyuan 030024, China; School of Power and Mechanical Engineering, Wuhan University, Wuhan 430072, China; Perception and Effectiveness Assessment for Carbon-neutrality Efforts, Engineering Research Center of Ministry of Education, Institute for Carbon Neutrality, Wuhan University, Wuhan 430072, China

**Keywords:** synergetic catalysis, CO_2_ hydrogenation, ethanol synthesis, controllable C–C coupling, C–O bond cleavage

## Abstract

CO_2_ catalytic hydrogenation presents a highly promising and alternative pathway for ethanol production, offering the dual benefits of large-scale CO_2_ emission reduction and green H_2_ storage. However, this approach faces major challenges such as low ethanol selectivity and activity, stemming from the complex reaction network and the lack of efficient catalyst design strategies. In this work, we proposed an effective catalyst design strategy for CO_2_ hydrogenation to ethanol by constructing synergetic catalytic sites between alloy nanoparticles and zeolite to decouple key competing reaction steps. The developed PdCu alloy catalyst encapsulated by Y zeolite achieved simultaneously high ethanol selectivity (>93%) and activity (>200 mg g_cat_^−1^ h^−1^), outperforming most previously reported heterogeneous catalysts. Mechanistic investigations revealed that the alloy–zeolite synergetic sites facilitate controllable C–C coupling and enhance C–O bond cleavage, thereby balancing selectivity and catalytic activity toward CO_2_ hydrogenation to ethanol. This work offers a feasible catalyst design strategy for the efficient conversion of CO_2_ into high-value-added and structurally complex products.

## Teaser

A Y zeolite encapsulated PdCu alloy catalyst with alloy-zeolite synergetic sites was developed for highly efficient ethanol synthesis from CO_2_ hydrogenation.

## INTRODUCTION

The increasing dependence on fossil fuels has led to substantial CO_2_ emissions, posing a serious threat to sustainable development. One promising approach to mitigate this issue is to close the carbon cycle by converting CO_2_ and green H_2_ into liquid fuels, including alcohols, gasolines, and aviation oils [1–4]. Among these, ethanol (C_2_H_5_OH) has received great attention due to its high energy density and low pollutant emission [5,6]. Furthermore, ethanol serves as an important solvent and disinfectant, playing a critical role in both daily life and industrial applications [7,8]. However, large-scale ethanol production currently depends on the fermentation of sugars derived from agricultural crops such as potatoes, corn, and sugarcane, which competes with global food supplies [7,8]. Thus, there is an urgent need to develop an alternative pathway for ethanol synthesis. Utilizing CO_2_ as a feedstock is particularly attractive, given its abundance, non-toxic nature, and renewability [9]. Nevertheless, the catalytic hydrogenation of CO_2_ to ethanol remains a major challenge, not only due to the thermodynamic stability and chemical inertness of CO_2_, but also because the reaction involves C–C bond formation while preserving specific C–O bond [7,10,11]. As a result, low ethanol selectivity and activity remain the primary obstacles to efficient CO_2_-to-C_2_H_5_OH conversion.

The molecular structure of ethanol implies that its synthesis from CO_2_ requires the integration of partial and complete C=O bond dissociations alongside C–C coupling, necessitating synergetic catalysis across multiple active sites. Two main reaction pathways have been proposed for CO_2_ hydrogenation to ethanol (Fig. S1). The first route proceeds via a CO intermediate, generated through the reverse water–gas shift reaction. The CO undergoes either dissociative or non-dissociative activation to form *CH*_x_* and *CO species, which then couple to form a *CH*_x_*CO intermediate and are further hydrogenated to C_2_H_5_OH [7,11]. This route is typically facilitated by Fischer–Tropsch metals such as Rh, Fe, and Co, but often suffers from uncontrollable C–C coupling, resulting in low ethanol selectivity (typically below 35%) [12–20]. The second route involves a CH_3_OH intermediate, produced via methanol synthesis. Methanol is dissociated to *CH_3_, which couples with *CO*_x_* to form *CH_3_CO*_x_*, followed by its further hydrogenation to C_2_H_5_OH [7,11,21,22]. This route allows more controllable C–C coupling and achieves high ethanol selectivity (often exceeding 90%), as demonstrated by single-atom catalysts such as Ir_1_-In_2_O_3_, Ir_1_-P*_x_*/In_2_O_3_, and Rh_1_/CeTiO*_x_* [23–25]. However, it faces challenges including a high energy barrier for C–O bond cleavage in CH_3_OH and competitive adsorption between H_2_ and CH_3_OH at the same metal sites, leading to limited ethanol activity (<100 mg g_cat_^−1^ h^−1^) [23–30]. Up to now, the performance of most catalysts for ethanol synthesis remains considerably inferior to those for methanol synthesis, underscoring the persistent difficulty in achieving efficient and selective CO_2_ conversion to multi-carbon products.

Zeolites have garnered significant attention in methanol conversion due to their well-defined Brønsted and Lewis acid sites and channel structures [31–33]. Metal@zeolite catalysts, which integrate metallic, acidic, and shape-selective functions, have been widely employed in key catalytic processes, including methane oxidation, selective hydrogenation, alkane dehydrogenation, syngas conversion, and CO_2_ hydrogenation [34–41]. By strategically combining metal-catalyzed H_2_ activation with zeolite-catalyzed CH_3_OH activation, it is expected that the competitive adsorption between H_2_ and CH_3_OH could be spatially decoupled, and the energy barrier for C–O bond cleavage in CH_3_OH be reduced, thereby enabling highly efficient CO_2_-to-ethanol conversion. However, such an approach had not been realized prior to this study.

In this work, we designed a Y zeolite-encapsulated PdCu alloy catalyst (PdCu@Y) for selective CO_2_ hydrogenation to ethanol. This architecture spatially separates H_2_ activation (on PdCu alloy sites) and CH_3_OH activation (on Y zeolite acid sites). Leveraging metal-zeolite synergetic catalysis (Fig. S1), the PdCu@Y catalyst achieves both controllable C–C coupling and facilitated C–O bond dissociation, resulting in simultaneously high ethanol selectivity (>93%) and activity (>200 mg g_cat_^−1^ h^−1^), which surpassed most previously reported heterogeneous catalysts for this reaction. The synergetic mechanism underlying ethanol formation was comprehensively elucidated through a combination of advanced characterization and theoretical calculations.

## RESULTS AND DISCUSSION

### Structural characterization of Y zeolite-encapsulated PdCu alloy catalyst

**Figure 1.**
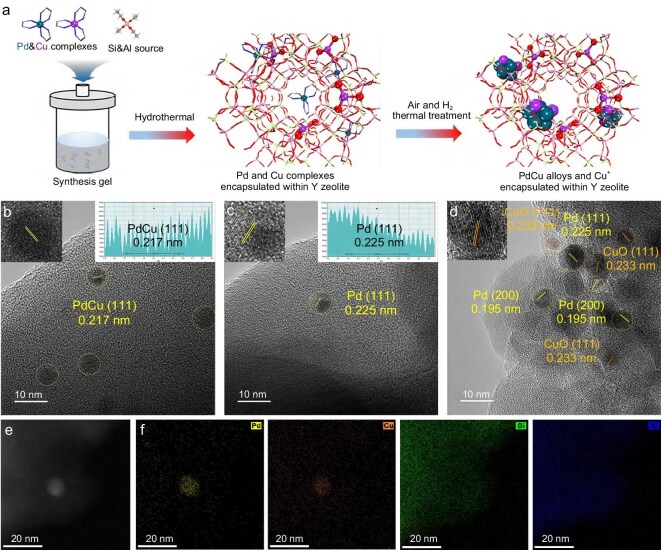
Synthesis and morphology characterization of series catalysts. (a) Schematic diagram for PdCu@Y catalyst synthesis. (b–d) HR-TEM of reduced PdCu@Y, Pd@Y, and PdCu/Y catalysts. (e) HAADF-STEM and corresponding (f) EDS mapping of single PdCu alloy nanoparticle on the reduced PdCu@Y catalyst.

The Y zeolite-encapsulated metal catalysts were synthesized via a ligand-protected hydrothermal method followed by H_2_ reduction. The Y zeolite-encapsulated PdCu alloy (PdCu@NaY) was chosen as an example to elucidate the synthetic process (Fig. 1a). Typically, PdCl_2_, CuCl_2_·2H_2_O, and excess ethylenediamine (EDA) were mixed in distilled water to form stable Pd and Cu complexes. EDA was employed to prevent premature precipitation of metal cations during the synthesis of Y zeolite [42]. Then, NaAlO_2_, NaOH, and colloidal silica were added to the aqueous solution, which was hydrothermally crystallized at 100°C for 2 days. After calcination and H_2_ reduction treatment, PdCu alloy nanoparticles along with Cu^+^ species were successfully encapsulated within the Y zeolite framework. The actual contents of Pd and Cu were shown in Table S1. X-ray diffraction (XRD) patterns confirmed the presence of the characteristic faujasite zeolite in all samples, with no detectable diffraction peaks assigned to Pd or Cu species (Fig. S2). The adsorption–desorption isotherms exhibited the standard type Ⅰ isotherms with BET surface area ranging between 700 and 800 m^2^ g^−1^ and a uniform pore size of ∼0.62 nm (Fig. S3 and Table S2). Further morphological analysis revealed that all samples consisted of microspheres ∼1–2 μm in diameter (Fig. S4). The above results collectively verified the successful synthesis of well-crystallized Y zeolite with encapsulated metal species.

The microstructure of the catalysts was further characterized by using transmission electron microscopy (TEM). High-angle annular dark field scanning transmission electron microscope (HAADF-STEM) and elemental mapping via energy-dispersive X-ray spectroscopy (EDS) revealed the atomic-scale dispersion of Pd and Cu in fresh PdCu@Y, with no discernible nanoparticles observed (Fig. S5). For reduced PdCu@Y (Fig. 1b), high-resolution TEM (HR-TEM) analysis identified an interplanar spacing of 0.217 nm, corresponding to the (111) plane of PdCu alloy. HAADF-STEM image displayed a uniform particle size distribution with an average diameter of 5.0 nm (Fig. S6). Elemental mapping further verified the overlapping distributions of Pd and Cu throughout the zeolite matrix (Fig. 1e and f, and Fig. S7). Collectively, these results strongly proved the successful encapsulation of PdCu alloy inside the Y zeolite. Pd@Y exhibited characteristic lattice spacings of 0.225 and 0.195 nm (Fig. 1c and Fig. S8), assigned to the (111) and (200) planes of metallic Pd. The Pd nanoparticles, with an average particle size of 5.0 nm, were also confirmed to be encapsulated within the Y zeolite (Figs S8 and S9). For Cu@Y, elemental mapping showed uniform dispersion of Cu species throughout the Y zeolite without detectable Cu or CuO nanoparticles (Fig. S10), implying that Cu cations were likely incorporated into the zeolite framework during hydrothermal synthesis [40]. In contrast, the supported PdCu/Y catalyst exhibited not only PdCu alloy and Pd nanoparticles, but also distinct CuO nanoparticles, identified by lattice fringes of 0.233 nm corresponding to the (111) planes of CuO (Fig. 1d), indicating severely phase-separated Pd and Cu species. Additionally, the majority of Pd and Cu nanoparticles in PdCu/Y were located on the external surface of the zeolite, with only limited alloying observed (Fig. S11), underscoring the critical role of Pd and Cu encapsulation within Y zeolite in promoting PdCu alloy formation. More importantly, the encapsulated PdCu alloy in PdCu@Y retained excellent structural stability even after the CO_2_ hydrogenation reaction, with no obvious structural degradation observed (Figs S12 and S13).

**Figure 2.**
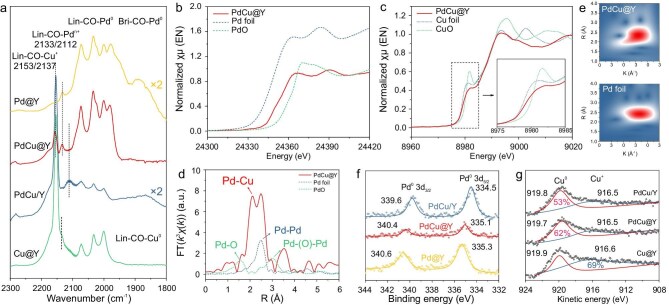
Electronic properties and coordination structure of Pd and Cu. (a) DRIFTS spectra of CO adsorption on Pd@Y, PdCu@Y, PdCu/Y, and Cu@Y after Ar purge for 5 min. (b) Pd K-edge XANES spectra of PdCu@Y, Pd foil, and PdO. (c) Cu K-edge XANES spectra of PdCu@Y, Cu foil, and Cu_2_O. (d) Fourier transform of *k*^3^-weighted EXAFS spectra of PdCu@Y, Pd foil, and PdO. (e) Wavelet transform of EXAFS spectra at Pd K-edge of PdCu@Y and Pd foil. XPS patterns of (f) Pd 3d and (g) Cu LMM of Pd@Y, PdCu@Y, PdCu/Y, and Cu@Y.

The electronic properties and coordination structures of Pd and Cu species were further investigated using diffuse reflectance infrared Fourier transform spectra of adsorbed CO (CO-DRIFTS), as shown in Fig. 2a and Fig. S14. Peaks above 2100 cm^−1^ were attributed to linear CO adsorption on cationic metal sites (M^*n*+^), those between 2000 and 2100 cm^−1^ to linear CO on metallic sites (M^0^), and bands below 2000 cm^−1^ to bridged CO adsorption on M^0^ [29,43]. For Cu@Y, both linear CO adsorption on Cu^+^ and Cu^0^ were detected, with the former dominating. The peaks at 2153/2137 cm^−1^ fitted well with adsorbed CO on Cu^+^-exchanged Y zeolite, indicating the formation of Cu^+^–O–Si bonds [44]. In Pd@Y, both linear and bridged CO adsorption on Pd^0^ were present, confirming the existence of Pd nanoparticles. Notably, the peak intensity of bridged CO adsorption decreased obviously on PdCu@Y, indicating a reduction in the contiguous Pd–Pd bonds due to the dilution effect of Cu and the formation of Pd–Cu bonds in the PdCu alloy [45]. In addition, linear CO adsorption on Cu^+^ was also detected in PdCu@Y, suggesting the presence of minor Cu^+^–O–Si bonds. Thus, the PdCu@Y sample contains both PdCu alloy and isolated Cu^+^ sites. In contrast, PdCu/Y exhibited pronounced signals for linear CO on Cu^+^ and bridged CO on Pd^0^, confirming the independent existence of Pd and Cu species with minimal PdCu alloy formation, consistent with TEM observations.

X-ray absorption spectroscopy (XAS) was performed to study the local coordination environment of PdCu@Y catalyst. For fresh sample (Fig. S15), the white line in the Pd K-edge X-ray absorption near-edge structure (XANES) spectra was close to that of PdO, indicating an average valence state of ∼+2. Fourier transform extended X-ray absorption fine structure (FT-EXAFS) revealed two distinct peaks at 1.5 and 2.5 Å, corresponding to Pd–O and Pd–Pd coordination shells [45]. The dominance of the Pd–O peak confirmed the high dispersion of Pd cations within the zeolite. Wavelet transform spectra analysis further supported this finding (Figs S15 and S16). For reduced PdCu@Y (Fig. 2b), the white line lay between those of Pd foil and PdO, suggesting an electron-deficient state of Pd species relative to Pd foil. Similarly, the Cu K-edge XANES spectra showed that the white line also lay between Cu foil and Cu_2_O (Fig. 2c), indicating an average valence state between 0 and +1 for Cu. The FT-EXAFS at the Pd K-edge showed two distinct peaks at 2.1 and 2.5 Å (Fig. 2d and Fig. S17), corresponding to Pd–Cu and Pd–Pd coordination shells, respectively [45], confirming the formation of PdCu alloy in PdCu@Y. Wavelet transform spectra analysis further supported these findings, revealing distinct contributions from both Pd–Cu and Pd–Pd coordination (Fig. 2e). Quantitative FT-EXAFS fitting yielded average coordination numbers (CN) of 1.57 for Pd–Cu and 5.07 for Pd–Pd (Table S3), which were significantly lower than the CN of 12 in Pd foil, consistent with the presence of small-sized PdCu alloy nanoparticles as observed by STEM. Notably, spent PdCu@Y exhibited XANES and FT-EXAFS features similar to those of the reduced sample, including the valence state as well as Pd–Cu and Pd–Pd coordination shells (Fig. S18), which confirmed the great stability of PdCu alloy under reaction conditions.

X-ray photoelectron spectroscopy (XPS) was used to further examine the electronic properties of the catalysts. The Pd 3d spectra of fresh PdCu@Y showed two characteristic peaks at binding energies of 336.3 and 341.6 eV (Fig. S19), assigned to 3d_5/2_ and 3d_3/2_ of Pd^2+^, confirming the atomic-scale dispersion of Pd cations within the zeolite, as previously evidenced by TEM and XAS [29]. For reduced and spent PdCu@Y, two new peaks emerged at 335.1 and 340.4 eV, which were 0.2 eV lower than those of Pd@Y (Fig. 2f and Fig. S19), consistent with the electron transfer from Cu to Pd upon PdCu alloy formation [45]. Furthermore, the binding energies of Pd 3d spectra in both PdCu@Y and Pd@Y were much higher than those in supported PdCu/Y, indicating strong electronic interactions between the metals and the zeolite when metals were encapsulated within the Y zeolite, which aligned with the higher Pd valence state on PdCu@Y observed in XANES spectra.

The Cu 2p spectra of reduced Cu@Y, PdCu@Y, and PdCu/Y are shown in Fig. S20. The binding energies in the ranges of 931.5–933.6 eV and 951.4–953.5 eV were attributed to Cu^0^ or Cu^+^ 2p_3/2_ and 2p_1/2_, respectively [40,43]. Cu@Y exhibited much higher binding energies (933.6 and 953.5 eV) compared to PdCu@Y and PdCu/Y, indicating stronger electronic interactions between Cu and the Y zeolite. Cu LMM Auger spectra further proved that Cu^+^ is the dominant species (69%) in Cu@Y (Fig. 2g), attributed to the formation of Cu^+^–O–Si bonds within the zeolite skeleton [40,44], which was consistent with the uniform Cu distribution observed by TEM and the Cu^+^ species detected in CO-DRIFTS. For PdCu@Y, the Cu 2p binding energies (931.9 and 951.8 eV) were higher than those of PdCu/Y (931.5 eV and 951.4 eV), supported by electronic transfer from Cu to Pd in the PdCu alloy [45]. Additionally, PdCu@Y exhibited a higher Cu^0^ content (62%) compared to Cu@Y (31%), indicating that Pd facilitated the reduction of Cu^2+^ via hydrogen spillover, in agreement with H_2_-TPR results (Fig. S21) [46]. In addition, a small amount of Cu species in PdCu@Y remained in the form of Cu^+^–O–Si bonds within the zeolite skeleton (Fig. 2g).

### Catalytic performance of CO_2_ hydrogenation to ethanol

**Figure 3.**
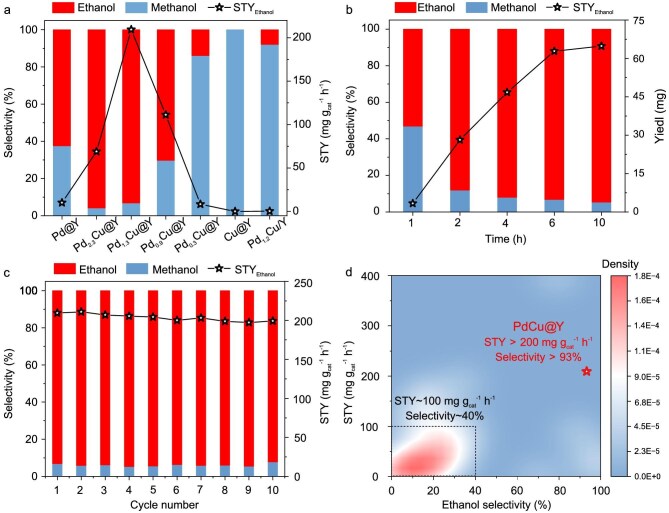
Catalytic performance of CO_2_ hydrogenation to ethanol. (a) Ethanol selectivity and STY on series catalysts. (b) Ethanol selectivity and yield changes vs. reaction time. (c) Catalytic stability test. (d) Performance comparison with the previously reported catalysts. Reaction conditions: 50 mg catalyst, 8 mL H_2_O, H_2_/CO_2_ = 3, 4 MPa, 200°C, 6 h.

The CO_2_ hydrogenation performance of the catalysts was evaluated in a tank reactor at 200°C and 4 MPa. Analysis of both gaseous and liquid products confirmed that only methanol and ethanol were formed (Fig. S22). As shown in Fig. 3a and Table S4, Pd@Y yielded an ethanol space time yield (STY) of 10.1 mg g_cat_^−1^ h^−1^ with 62.4% selectivity and CO_2_ conversion of 0.64%. The introduction of Cu significantly enhanced CO_2_ conversion, ethanol STY and selectivity, with these metrics following volcano-shaped trends as a function of the Pd/Cu molar ratio. Among all tested catalysts, Pd_1.3_Cu@Y showed the highest ethanol STY of 209.3 mg g_cat_^−1^ h^−1^ with 93.2% selectivity and CO_2_ conversion of 8.98%. In contrast, Cu@Y produced only methanol, underscoring the essential role of PdCu alloy in enabling ethanol formation [44]. A comparison between encapsulated Pd_1.3_Cu@Y and supported Pd_1.2_Cu/Y catalysts with comparable metal loadings further highlighted the superiority of the encapsulated structure. The supported catalyst exhibited minimal activity for ethanol synthesis, with an STY of only 0.2 mg g_cat_^−1^ h^−1^ and selectivity of 7.9%. These results clearly demonstrated that the encapsulated structure was crucial for high ethanol yield and selectivity, indicating strong synergetic effect between the PdCu alloy and the Y zeolite in selective hydrogenation of CO_2_ to ethanol.

The effects of reaction conditions on CO_2_ hydrogenation to ethanol were evaluated using the optimized Pd_1.3_Cu@Y (denoted as PdCu@Y) catalyst. As shown in Fig. 3b, both ethanol yield and selectivity were initially low (3.4 mg g_cat_^−1^ and 53.1%) but increased steadily with time, reaching 64.9 mg g_cat_^−1^ and 94.7% after 10 h reaction. In contrast, methanol selectivity decreased progressively over the same period. Additional tests under varying temperatures and pressures (Fig. S23) revealed that higher temperature and pressure promoted ethanol formation, indicating that the synthesis of ethanol was kinetically controlled [23]. Furthermore, low ethanol STY was consistently accompanied by high methanol selectivity across all reaction conditions, suggesting that methanol acted as a key intermediate in the conversion of CO_2_ hydrogenation to ethanol [7,11].

The stability of PdCu@Y was evaluated in ten consecutive reaction cycles at 200°C and 4 MPa. As shown in Fig. 3c, the catalyst exhibited great stability, with ethanol STY exceeding 200 mg g_cat_^−1^ h^−1^ and ethanol selectivity being ∼94%. Since ethanol has a higher economic value compared to methanol, the selective conversion of CO_2_ to ethanol has attracted significant research interest within catalytic CO_2_ utilization [4,7,32]. However, most existing catalysts still fall short in performance relative to methanol synthesis benchmarks, typically achieving ethanol selectivity and STY values around 40% and 100 mg g_cat_^−1^ h^−1^, respectively (Fig. 3d). In contrast, the designed Y zeolite-encapsulated PdCu alloy catalyst developed in this work delivered obviously superior performance, with ethanol selectivity and STY reaching 93% and 200 mg g_cat_^−1^ h^−1^, outperforming the majority of previously reported heterogeneous catalysts (Fig. 3d and Table S5).

### Chemisorption and acid properties

The excellent catalytic performance of PdCu@Y was strongly related to its unique structural features and the synergy interaction between the PdCu alloy and the Y zeolite. To elucidate this structure–performance relationship, we selected encapsulated PdCu@Y, Pd@Y, Cu@Y, and supported PdCu/Y samples for comparative study. A series of characterizations were first conducted to examine the chemisorption behavior and acid properties of the reduced samples.

**Figure 4.**
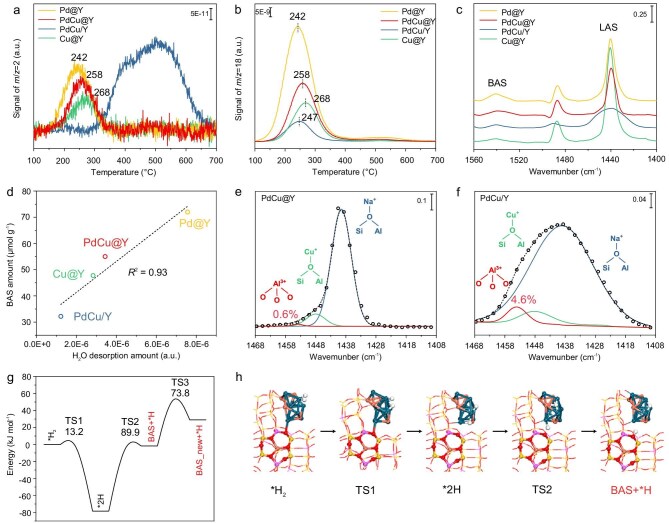
Chemisorption and acid properties of samples. (a) H_2_ and (b) H_2_O signals in H_2_-TPD. (c) Pyridine adsorption IR spectra of reduced samples. (d) Correlation of the BAS amount with H_2_O desorption amount in H_2_-TPD. LAS peak splits of (e) encapsulated PdCu@Y and (f) supported PdCu/Y samples. (g) Reaction energy profiles and corresponding (h) structural models of H_2_ dissociation and BAS formation on PdCu@Y catalyst. Pd (blue), Cu (orange), Si (yellow), Al (pink), O (red), and H (white).

The H_2_ temperature-programmed desorption (H_2_-TPD) results are shown in Fig. 4a. The encapsulated and supported samples showed markedly different H_2_ desorption behaviors: the encapsulated samples (Pd@Y, PdCu@Y, and Cu@Y) displayed H_2_ desorption peak around 250°C (Fig. 4a), whereas the supported PdCu/Y displayed a broad H_2_ desorption peak between 300 and 700°C, with a H_2_ desorption amount 6.3 times greater than that of PdCu@Y (Table S6). Given the comparable metal loading and nanoparticle size between PdCu@Y and PdCu/Y, the observed discrepancy suggested that active *H species may desorb in alternative forms.

Consequently, we detected H_2_O signal during H_2_-TPD. As shown in Fig. 4b, significant H_2_O desorption was detected from all samples. The H_2_O desorption temperatures for Pd@Y, PdCu@Y, and Cu@Y were 242, 258, and 268°C, respectively, which were exactly same with H_2_ desorption temperatures. Moreover, the H_2_O desorption peak areas followed the order Pd@Y > PdCu@Y > Cu@Y, consistent with H_2_ desorption peak area (Table S6). These results indicated that on encapsulated catalysts, active *H species derived from H_2_ dissociation on metal surface spilled over to the zeolite, forming hydroxyl groups that subsequently desorbed as H_2_O. In contrast, the supported PdCu/Y exhibited mismatched H_2_ and H_2_O desorption peaks and a much smaller H_2_O signal, confirming the absence of effective hydrogen spillover. Based on the peak areas of H_2_ and H_2_O, the H_2_ activation ability followed the order Pd > PdCu alloy > Cu, highlighting the role of Pd in promoting H_2_ activation and the weakened hydrogenation ability resulting from PdCu alloy formation [45,47].

According to H_2_-TPD results, active *H species derived from H_2_ dissociation on metal sites could spill over to the zeolite, forming hydroxyl groups that might act as Brønsted acid sites (BAS). To investigate the acid properties, pyridine adsorption infrared spectra (IR) was performed. As shown in Fig. 4c, the absorption peaks at 1540 and 1440 cm^−1^ were ascribed to pyridine adsorbed on BAS and Lewis acid sites (LAS), respectively [48]. The supported PdCu/Y exhibited the lowest BAS amount (31.0 μmol g^−1^) among all samples (Table S6). Moreover, a strong linear relationship was observed between the amount of H_2_O desorption in H_2_-TPD and the BAS amount (Fig. 4d), confirming that hydrogen spillover from the metal to the zeolite promoted the formation of hydroxyl groups acting as BAS. For the encapsulated samples, the BAS amount followed the order Pd@Y (75.1 μmol g^−1^) > PdCu@Y (55.0 μmol g^−1^) > Cu@Y (47.8 μmol g^−1^), aligning with the trend in H_2_ activation ability of the metals. Although supported PdCu/Y had metal contents similar to those of encapsulated PdCu@Y, the weak metal-zeolite interaction inhibited the spillover of active *H species, resulting in the lowest BAS amount. The LAS characteristic peaks of PdCu@Y and PdCu/Y are shown in Fig. 4e and f. The peaks at 1454, 1448, and 1440 cm^−1^ were attributed to coordinatively unsaturated Al^3+^, and Cu^+^ and Na^+^ bonded to the zeolite skeleton, respectively [48]. The presence of Cu^+^-related LAS in PdCu@Y confirmed the existence of Cu^+^–O–Si species, consistent with XPS and CO-DRIFTS results. In addition, the fraction of coordinatively unsaturated Al^3+^ of PdCu@Y (0.6%) was much less than that of PdCu/Y (4.6%), indicating that the synergy between the alloy and the zeolite in encapsulated catalysts promoted the conversion of LAS (unsaturated Al^3+^) to BAS (Si–OH–Al).

The dissociation of H_2_ and the formation of BAS were also studied from a theoretical standpoint. As shown in Fig. 4g, H_2_ dissociation readily occurred on the PdCu alloy with a low energy barrier (*E*_a_) of only 13.2 kJ mol^−1^, and was highly exothermic, with a reaction energy (Δ*E*) of 78.4 kJ mol^−1^. The resulting active *H species subsequently spilled over from the PdCu alloy to nearby Si–O–Al motifs located on the 6-membered rings of Y zeolite, leading to the generation of BAS (Fig. 4h). This spillover process required an *E*_a_ of 89.9 kJ mol^−1^ with Δ*E* of 76.7 kJ mol^−1^. Moreover, the proton of the BAS can diffuse across the 6-membered ring with an *E_a_* of 73.8 kJ mol^−1^, which could facilitate its participation in subsequent CO_2_ hydrogenation reactions (Fig. 4g and Fig. S24). These calculated results supported the existence of a synergetic effect between the metal alloy and the zeolite.

### Alloy-zeolite synergetic mechanism

**Figure 5.**
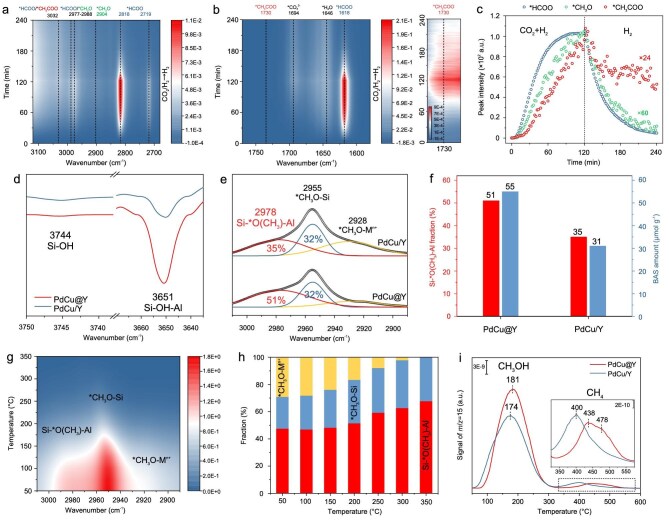
Reaction mechanism study of CO_2_ hydrogenation to ethanol. (a and b) *In-situ* DRIFTS of CO_2_ hydrogenation at 0.1 MPa and 200°C, and (c) dynamic IR peak intensity changes vs. reaction time on PdCu@Y catalyst. *In-situ* DRIFTS of (d) CH_3_OH adsorption and corresponding (e) peak split results on PdCu@Y and PdCu/Y. (f) Si–*O(CH_3_)–Al fractions and BAS amounts of PdCu@Y and PdCu/Y. (g) *In-situ* DRIFTS of CH_3_OH-TPD and corresponding (h) *CH_3_O species fractions on PdCu@Y. (i) Signals of CH_3_OH and CH_4_ (*m/z* = 15) in CH_3_OH-TPSR under H_2_ atmosphere on PdCu@Y and PdCu/Y.

To reveal the reaction mechanism of CO_2_ hydrogenation to ethanol, we performed *in-situ* DRIFTS test under conditions of 0.1 MPa and 200°C on PdCu@Y (Fig. 5a and b). The peak assignment was summarized in Table S7. Upon introduction of the CO_2_/H_2_ mixture, the peaks assigned to carbonate (*CO_3_^2−^) and formate (*HCOO) appeared and intensified immediately, indicating the rapid CO_2_ adsorption and hydrogenation on the catalyst surface [19,25]. As the reaction progressed, the emergence of a methoxy (*CH_3_O) suggested further hydrodeoxygenation of *HCOO [19,25]. Subsequently, acetate (*CH_3_COO) species—a key intermediate in ethanol synthesis—was observed, likely formed via coupling between *CH_3_ and *CO_2_ species [49,50] Notably, no adsorbed or gaseous CO was detected (Fig. S25) [29,43], ruling out the involvement of *CO intermediate in the ethanol formation pathway on PdCu@Y—a point of ongoing debate in CO_2_-to-ethanol mechanisms [7,11]. The evolution of key intermediates (*HCOO, *CH_3_O, and *CH_3_COO) during CO_2_ hydrogenation was further compared in Fig. 5c. Among these species, the peak intensity of *HCOO increased most rapidly, indicating that CO_2_ was initially converted into *HCOO. The increasing rate of *CH_3_O was moderate, while that of *CH_3_COO was the slowest. The formation sequence followed the order *HCOO > *CH_3_O > *CH_3_COO, supporting a methanol-mediated *CH_3_–*CO_2_ coupling mechanism. As the reaction pressure was increased to 1 MPa (Fig. S26), the conversion rates from *HCOO to *CH_3_O and subsequently to *CH_3_COO were obviously enhanced. Moreover, an ethoxy (*C_2_H_5_O) intermediate was detected as the reaction proceeded, suggesting hydrogenation of *CH_3_COO. These results collectively demonstrated that ethanol formation on PdCu@Y proceeded through stepwise hydrogenation of CO_2_ to *HCOO and *CH_3_O, subsequent *CH_3_O dissociation to *CH_3_, *CH_3_–*CO_2_ coupling to form *CH_3_COO, and final hydrogenation to *C_2_H_5_O and ethanol. This methanol-mediated mechanism was crucial for achieving controllable C–C coupling and high ethanol selectivity over PdCu@Y catalyst.

As for PdCu/Y and Pd@Y catalysts, similar intermediates and CO_2_ conversion pathway were observed as with PdCu@Y, confirming a consistent reaction mechanism for CO_2_ hydrogenation (Figs S27 and S28). However, the signal intensity of the key *CH_3_COO intermediate was significantly weaker on PdCu/Y and Pd@Y compared to PdCu@Y, correlating with their lower ethanol synthesis activity (Fig. S29). Notably, Pd@Y showed much faster formation and conversion rates of *HCOO and *CH_3_O intermediates than PdCu@Y, which could be attributed to the stronger hydrogenation ability of Pd (Fig. S29). This implied that on Pd@Y, hydrogenation reactions occurred more rapidly than C–C coupling reaction, thereby hindering the formation of *CH_3_COO intermediate. In contrast, the supported PdCu/Y catalyst showed the lowest peak intensities and the slowest conversion rates among all intermediates, highlighting its inferior performance. These results underscored the critical importance of the synergetic catalysis between the alloy and the zeolite in promoting efficient CO_2_ hydrogenation toward ethanol.

*In-situ* DRIFTS analysis revealed that the *CH_3_O intermediate served as the source of *CH_3_ in the *CH_3_COO formation pathway. The C–O bond cleavage of methanol generally encountered the problem of high energy barrier, resulting in low catalytic activity in CO_2_ hydrogenation to ethanol [7,11]. To elucidate the methanol activation and C–O dissociation mechanism, we compared PdCu@Y and PdCu/Y catalysts using *in-situ* DRIFTS. Upon methanol vapor introduction, consumption of hydroxyl species (Si–OH and Si–OH–Al) was observed, indicating the involvement of zeolite surface hydroxyl groups in methanol adsorption and activation (Fig. 5d). Characteristic *CH_3_O peaks appeared at 2978, 2955, and 2928 cm^−1^ (Fig. 5e), corresponding to methanol adsorbed on BAS (Si–OH–Al), forming Si–*O(CH_3_)–Al, silanol groups (Si–OH, forming *CH_3_O–Si), and metal sites (M^*n*+^, forming *CH_3_O–M^*n*+^), respectively [44,51]. Quantification revealed that Si–*O(CH_3_)–Al species dominated (51%) in PdCu@Y, consistent with its abundant BAS, whereas PdCu/Y exhibited a lower proportion (35%) of such species (Fig. 5f).

TPD further evaluated methanol adsorption strength. As the temperature increased from 50 to 350°C, the *CH_3_O–M^*n*+^ species decreased quickly on PdCu@Y, while the Si–*O(CH_3_)–Al and *CH_3_O–Si species desorbed more slowly (Fig. 5g). At 350°C, the *CH_3_O–M^*n*+^ species completely disappeared, only Si–*O(CH_3_)–Al and *CH_3_O–Si species remained (Fig. 5h). Notably, the fraction of Si–*O(CH_3_)–Al species increased significantly from 47% to 68%, and *CH_3_O–Si rose slightly from 23% to 32%, indicating stronger methanol adsorption on Si–OH–Al sites (BAS). This enhanced adsorption facilitates C–O bond dissociation to form *CH_3_ intermediates. Therefore, the encapsulated PdCu@Y catalyst exhibited superior C–O bond cleavage capability compared to the supported PdCu/Y catalyst, which was crucial for overcoming the intrinsic activity limitations in CO_2_-to-ethanol conversion.

To further evaluate methanol conversion behavior, we conducted methanol temperature-programmed surface reaction (CH_3_OH-TPSR) experiment under H_2_ atmosphere over PdCu@Y and PdCu/Y catalysts. As shown in Fig. 5i and Fig. S30, the main desorption peak around 180°C was assigned to weakly adsorbed methanol on the zeolite. Notably, a broad CH_4_ signal appeared between 350 and 550°C for both PdCu@Y and PdCu/Y, indicating CH_4_ formation from C–O bond cleavage in methanol followed by hydrogenation of the resulting *CH_3_ intermediate—a known side reaction in CO_2_-to-ethanol conversion [7,11].

The CH_4_ desorption temperature increased from 400°C on PdCu/Y to 438°C and 479°C on PdCu@Y, indicating effective suppression for CH_3_OH conversion to CH_4_ on PdCu@Y. Although CH_3_OH-TPD DRIFTS confirmed that PdCu@Y exhibited better C–O bond dissociation ability, the higher CH_4_ desorption temperature suggested that *CH_3_ hydrogenation was inhibited, thereby promoting the desired *CH_3_–*CO_2_ coupling pathway. In summary, the BAS played a critical role in the C–O bond cleavage of *CH_3_O to form *CH_3_ intermediate, and the *CH_3_–*CO_2_ coupling was favored over *CH_3_ hydrogenation to CH_4_ on PdCu@Y, which were key factors enabling highly active and selective ethanol synthesis from CO_2_ hydrogenation.

**Figure 6.**
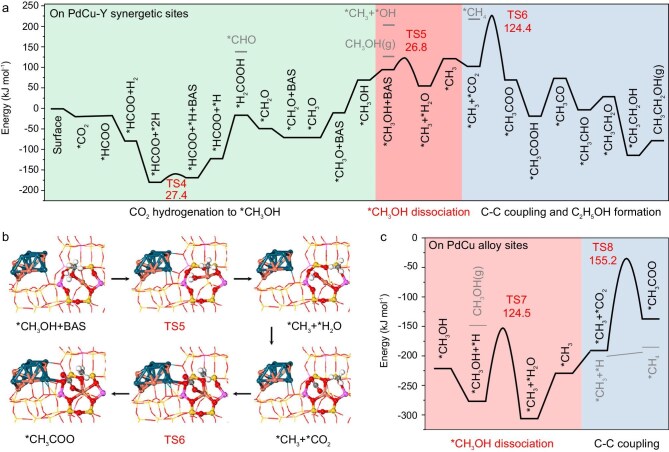
Theoretical perspective of alloy-zeolite synergetic mechanism. (a) DFT-calculated reaction energy profile and (b) key structural models on PdCu–Y synergetic sites. (c) Reaction energy profile of *CH_3_OH dissociation and *CH_3_–*CO_2_ coupling steps on PdCu alloy sites. Pd (blue), Cu (orange), Si (yellow), Al (pink), C (gray), O (red), and H (white).

To further reveal the synergetic mechanism between the alloy and zeolite in CO_2_ hydrogenation to ethanol, we investigated the underlying reaction mechanism by DFT calculations. A PdCu@Y model was constructed by introducing a Cu^+^ cation in the 6-membered ring and a Pd_9_Cu_4_ cluster within the 12-membered ring channel of Y zeolite (see Supporting Information for details). The overall process involved three key stages: CO_2_ hydrogenation to *CH_3_OH, dissociation of *CH_3_OH to *CH_3_, and the C–C coupling followed by hydrogenation to C_2_H_5_OH [24]. The reaction energy profile is shown in Fig. 6a (with structural models provided in Fig. S31). CO_2_ was adsorbed at the Cu^+^ sites within the 6-membered rings of Y zeolite with an adsorption energy (Δ*E*) of −19.7 kJ mol^−1^. The adsorbed *CO_2_ was hydrogenated to form *HCOO with a low reaction energy of 2.6 kJ mol^−1^. Interestingly, the presence of adsorbed *HCOO intermediates facilitated H_2_ dissociation (Δ*E* = −100.8 kJ mol^−1^) and BAS formation (Δ*E* = 11.4 kJ mol^−1^, *E*_a_ = 27.4 kJ mol^−1^). Given this promoting effect, protons were directly introduced in subsequent steps to represent BAS without recalculating H_2_ dissociation and *H transfer. Further hydrogenation of *HCOO yielded *HCOOH intermediate (Δ*E* = 46.5 kJ mol^−1^), which preferentially underwent a two-step pathway involving hydrogenation and deoxygenation to form *CH_2_O intermediate (Δ*E* = 106.1 and −33.5 kJ mol^−1^), rather than direct hydrodeoxygenation to produce *CHO (Δ*E* = 263.4 kJ mol^−1^). *CH_2_O was then readily hydrogenated to *CH_3_OH adsorbed at the Cu^+^ sites.

Critically, nearby BAS effectively promoted the dissociation of *CH_3_OH to *CH_3_ with concurrent release of H_2_O (Fig. 6b), requiring an *E*_a_ of only 26.8 kJ mol^−1^ and being exothermic by 40.9 kJ mol^−1^. In contrast, direct dissociation of *CH_3_OH to *CH_3_ and *OH was highly unfavorable (Δ*E* = 120.8 kJ mol^−1^). These results aligned with *in-situ* DRIFTS observations of abundant Si–*O(CH_3_)–Al species on PdCu@Y (Fig. 5e), again confirming the important role of BAS in facilitating *CH_3_OH dissociation to produce *CH_3_ intermediate. Such Si–*O(CH_3_)–Al configuration also inhibited undesired methanation, which had a high energy cost (Δ*E* = 120.3 kJ mol^−1^). Moreover, this dissociation released the Cu^+^ site, allowing it to adsorb another CO_2_ molecule for the subsequent *CH_3_–*CO_2_ coupling step (Fig. 6b). This coupling formed *CH_3_COO intermediate—the rate-determining step of the overall pathway (*E*_a_ = 124.4 kJ mol^−1^, Δ*E* = −33.2 kJ mol^−1^). Hydrogenation of *CH_3_COO to *CH_3_CH_2_OH was generally favorable, with the hydrogenation of *CH_3_COO itself being the most energy demanding step (Δ*E* = 92.4 kJ mol^−1^). Notably, the *CH_3_COO intermediates bonded to Cu^+^ sites can migrate to adjacent PdCu alloy sites (Δ*E* = −43.1 kJ mol^−1^), which further facilitated the following hydrogenation (Figs S32 and S33).

The reaction energy profile was also examined on PdCu alloy sites alone (that is, without involving zeolite sites). CO_2_ can strongly adsorb at the Pd–Pd sites of PdCu alloy, while its weak adsorption occurred on Pd–Cu sites (Fig. S34, corresponding structural models are shown in Fig. S35). The adsorbed *CO_2_ readily underwent continuous hydrodeoxygenation steps to form the key *CH_3_OH intermediate. However, in the absence of BAS, the C–O bond dissociation of *CH_3_OH on PdCu alloy was significantly hindered, requiring an *E*_a_ of 124.5 kJ mol^−1^—markedly higher than that on the PdCu–Y synergistic sites (26.8 kJ mol^−1^; Fig. 6c). Furthermore, the resulting *CH_3_ intermediate favored hydrogenation to CH_4_ (Δ*E* = 2.5 kJ mol^−1^) rather than its coupling with *CO_2_ to form *CH_3_COO, which presented a higher energy cost (Δ*E* = 54.2 kJ mol^−1^, *E*_a_ = 155.2 kJ mol^−1^). These computational results aligned with the lower CH_4_ desorption temperature observed in CH_3_OH-TPSR experiments for PdCu/Y (Fig. 5i). Together, these findings underscored the crucial role of alloy-zeolite synergy in enabling efficient *CH_3_OH dissociation to *CH_3_ and facilitating the subsequent *CH_3_–*CO_2_ coupling reaction toward ethanol.

In summary, mechanism study revealed a strong synergetic effect between PdCu alloy and acid sites in promoting *CH_3_O dissociation to *CH_3_, a critical step in ethanol formation. Meanwhile, the deep hydrogenation of *CH_3_ was suppressed, thus favoring *CH_3_–*CO_2_ coupling to form key *CH_3_COO intermediate. These two factors collectively accounted for the superior ethanol synthesis performance over PdCu@Y. In contrast, the other catalysts lacked such alloy-acid synergy or exhibited either overly strong or insufficient hydrogenation ability, resulting in low ethanol selectivity and poor catalytic activity.

## CONCLUSIONS

In this work, a Y zeolite-encapsulated PdCu alloy catalyst (PdCu@Y) was designed and synthesized for CO_2_ hydrogenation to ethanol, demonstrating superior catalytic performance with high ethanol selectivity (>93%) and activity (>200 mg g_cat_^−1^ h^−1^), which surpassing most previously reported heterogeneous catalysts. Both experimental and theoretical results conclusively illustrated the synergetic catalysis between the PdCu alloy and the zeolite. The confinement effect of the zeolite first ensured the formation of a uniform PdCu alloy, while the encapsulated PdCu alloy promoted the formation of BAS in zeolite under H_2_ atmosphere. Mechanism study further revealed that the reaction followed a methanol-mediated route, wherein BAS played a critical role in facilitating the C–O bond cleavage of methanol—a recognized pivotal step in ethanol synthesis. As a result, the synergistic alloy-zeolite catalytic sites enabled both controllable C–C coupling and enhanced C–O bond cleavage, thereby achieving an optimal balance between high selectivity and activity. This work provided a promising catalyst design strategy for the efficient synthesis of high-value-added products and the decoupling of complex reaction networks.

## MATERIALS AND METHODS

The Y zeolite-encapsulated metal catalysts were synthesized via a ligand-protected hydrothermal method followed by H_2_ reduction. The detailed synthesis, characterization and catalytic test methods were available in supplementary data.

## Supplementary Material

nwag303_Supplemental_File
