# Supplementary material for: Synergetic catalysis via zeolite-encapsulated PdCu alloy for highly efficient CO2 hydrogenation to ethanol
Source: Natl Sci Rev. 2026 May 23;13(15):nwag303. doi: 10.1093/nsr/nwag303 (PMC13463630; doi:10.1093/nsr/nwag303)
Supplement: Supplementary file 1 [file nwag303_supplemental_file.pdf]

Synergetic catalysis via zeolite-encapsulated PdCu alloy for highly efficient CO_2 hydrogenation to ethanol

Di Xu,[1#]* Shaojia Song,[3#] Guoqiang Hou,[1] Ruosong He,[1] Haifeng Fan,[1] Yangyang Li,[1] Siyi Huang,[1] Hanzhu Zhang,[1] Baojun Wang,[3] Riguang Zhang,[3]* Mingyue Ding[1,2]*

[1] School of Power and Mechanical Engineering, Wuhan University; Wuhan, 430072, China
[2] Perception and Effectiveness Assessment for Carbon-neutrality Efforts, Engineering Research Center of Ministry of Education, Institute for Carbon Neutrality, Wuhan University; Wuhan, 430072, China
[3] State Key Laboratory of Clean and Efficient Coal Utilization, College of Chemistry and Chemical Engineering, Taiyuan University of Technology; Taiyuan, 030024, China

*Correspondence author: x_d@whu.edu.cn, zhangriguang@tyut.edu.cn, dingmy@whu.edu.cn.
[#]These authors contributed equally to this work.

Experimental section

Catalyst synthesis

Y zeolite was synthesized by a hydrothermal method. Typically, 0.7 g $NaAlO_2$ and 4.15 g NaOH were dissolved in 50 mL deionized water. Then 9.62 mL 40% colloidal silica was added into above solution and stirred for 6 h. The mixture was then transferred into a Teflon-lined stainless-steel autoclave and dynamically crystallized at 100 ºC for 2 days. The products were thoroughly washed with deionized water and dried at 100 ºC overnight. The sample was then calcinated at 550 ºC for 6 h.

The synthesis of PdCu@Y was the same as Y except that $PdCl_2$, $CuCl_2·2H_2O$ and ethylenediamine (EDA) were firstly dissolved in 50 mL deionized water. The molar ratio of EDA and metal were fixed at 250. The input quantity of metal salt and actual contents of Pd and Cu, determined by ICP-MS, were showed in Table S1.

PdCu/Y was prepared by impregnating 14.0 mg $PdCl_2$, 10.7 mg $CuCl_2·2H_2O$ and EDA aqueous solution (1.18 g EDA dissolved in 5 mL deionized water) to 2 g Y zeolite, followed by drying and calcinating at the same conditions.

Catalyst characterization

Inductively coupled plasma optical emission spectrometer (ICP-OES) analysis was carried out on Agilent 5110 to measure the metal content in catalysts. The N_2 adsorption-desorption was tested on a 100B (JWBK) instrument. X-ray diffraction (XRD) was performed on an Ultima IV X-Ray Diffractometer using Cu Kα radiation. The sample was scanned from 5-80º with a scanning rate of 10º min^{-1}. Scanning electron microscopy (SEM) measurement was performed on a Zeiss SIGMA field emission scanning electron microscope. Transmission Electron Microscopy (TEM) measurement was performed on a JEM-2100F (JEOL) microscope. Energy dispersive X-ray spectroscopy (EDS) mapping was carried out on a Technai G2 F30 (FEI) microscope. X-ray photoelectron spectroscopy (XPS) experiment was performed on a Thermo Fisher Scientific ESCALABXi$^+$ XPS spectrometer with Al Kα radiation. Gas chromatography mass spectrometry (GC-MS) analysis was performed on an Agilent 8890 GC-7000E system. XAFS spectra were collected at the BL11B beamline of the Shanghai Synchrotron Radiation Facility (SSRF). During the measurement, the synchrotron radiation ring was operated at 3.5 GeV and the electron current was 200 mA in the top-up mode.

H_2 temperature-programmed reduction (H_2-TPR) experiment was performed on a Micromeritics ASAP 2930. 50 mg spent sample was used in each test. The detailed procedure was described as follows. First, the spent catalyst was treated under Ar flow (30 mL min^{-1}) at 400 ºC for 0.5 h and cooled down to 50 ºC. Then, the gas was switched to 10% H_2/Ar at a flow rate of 50 mL min^{-1} for 30 min. After that, the TPR measurement was conducted from 50 ºC to 800 ºC (10 ºC min^{-1}) with a thermal conductivity detector.

H_2 temperature-programmed desorption (H_2-TPD) experiment was performed on a Micromeritics ASAP 2930. 50 mg spent sample was used in each test. The detailed procedure was described as follows. First, the spent catalyst was treated under Ar flow (30 mL min^{-1}) at 400 ºC for 0.5 h and cooled down to 50 ºC. After a pre-adsorption of H_2 at 50 ºC, the gas was switched to Ar at a flow rate of 50 mL min^{-1} to purge the

residual H_2 for 30 min. After that, the TPD measurement was conducted from 50 ºC to 800 ºC (10 ºC min^{-1}) with a quadrupole mass spectrometer (MS, Hidden, QIC-20) detector. The signals of m/z of 2 and 18 were detected.

CH_3OH temperature-programmed surface reaction (CH_3OH-TPSR) experiment was performed on a Micromeritics ASAP 2930. 50 mg spent sample was used in each test. The detailed procedure was described as follows. First, the spent catalyst was treated under Ar flow (30 mL min^{-1}) at 400 ºC for 0.5 h and cooled down to 50 ºC. After a pre-adsorption of CH_3OH at 50 ºC, the gas was switched to Ar at a flow rate of 50 mL min^{-1} to purge the residual H_2 for 30 min. After that, the TPSR measurement was conducted under H_2/Ar flow from 50 ºC to 600 ºC (10 ºC min^{-1}) with a quadrupole mass spectrometer (MS, Hidden, QIC-20) detector. The signals of m/z of 31 and 15 were detected.

In-situ diffuse reflection infrared Fourier transformation spectroscopy (DRIFTS) measurement was accomplished in a reaction cell on a Nicolet iS50 FT-IR spectrometer with a MCT detector. The sample was firstly reduced at 400 ºC in pure H_2 flow of 30 mL min^{-1} for 2 h, and then purged with Ar at the same temperature for 1 h. The background spectra were collected by 32 scans at a resolution of 4 cm^{-1} after cooling down to desired temperatures in an Ar flow of 30 mL min^{-1}. CO adsorption test was performed at 30 ºC in a CO/Ar flow for 0.5 h and then switched to Ar flow for 1 h. CO_2 hydrogenation test was carried out at 200 ºC in a H_2/CO_2 flow of 30 mL min^{-1} for 2 h and then switched to H_2 flow for 2 h at desired pressures. CH_3OH adsorption test was performed at 50 ºC in a CH_3OH/Ar flow for 0.5 h, switched to Ar flow for 0.5 h and then increased to 350 ºC (5 ºC min^{-1}) for 0.5 h.

Pyridine adsorption infrared spectroscopy (IR) test was accomplished in an *in-situ* reaction cell on a Bruker Tensor 27 spectrometer. The sample was firstly vacuumed to 10^{-3} Pa at 350 ºC for 1 h to clean the surface. Then the background spectra were collected by 32 scans at a resolution of 4 cm^{-1} after cooling down to 30 ºC. The pyridine adsorption was carried out under pyridine vapor at 30 ºC for 0.5 h. After that, the reaction cell was vacuumed to 10^{-3} Pa again at 200 ºC for 0.5 h to eliminate the weakly adsorbed pyridine. And the IR spectra were collected after cooling down to 30 ºC.

Catalytic test

CO_2 hydrogenation test was performance in a 100 mL stainless steel autoclave reactor. Typically, catalyst was first reduced at 400 ºC in pure H_2 for 2 h. Then 50 mg reduced catalyst was placed into the reactor with 8 mL deionized water. Before reaction, the reactor was purged with feed gas (H_2/CO_2=3) several times to remove air, then pressurized up to 4 MPa at room temperature. The reactor was heated to 200 °C and stirred at 600 rpm for 6 h. The gaseous products were analyzed by a gas chromatograph (Ruimin GC2060) equipped with Porapak Q and 5A MolSieve packed columns for $H_2/N_2/CO/CH_4/CO_2$ analysis via thermal conductivity detector. The liquid products were analyzed by a gas chromatograph with dioxane as an internal standard substance. The product selectivity was based on the moles of carbon. The catalytic tests were repeated three times for each catalyst.

DFT calculation method

The spin-polarized density function theory (DFT) calculations were implemented in VASP 5.4.4 package with using the GGA-PBE electron exchange-correlation function. The grimme's D3 method was employed to correct the van der Waals dispersion interactions in all calculations. Brillouin zone sampling was Gamma point. The cutoff energy and force criterion was set as 400 eV and 0.05 eV/Å, respectively. Optimized structures were obtained when both total energy change and maximum forces on all ions were less than 10^{-5} eV and 0.05 eV/Å, respectively. The transition state searches were conducted using the combined nudged elastic band (CI-NEB) and improved dimer method (IDM), and were confirmed by only one imaginary vibrational frequency along the reaction coordinate.

Y zeolite belongs to the faujasite (FAU) framework, a cubic structure in the space group $Fd\overline{3}m$. The pure silica polymorph of this framework contains 576 atoms per unit cell (occupying 192 tetrahedral, T-sites). Introducing Al, protons, and PdCu into the structure at realistic ratios, the contents of the unit cell would exceed 600 atoms. This $Fd\overline{3}m$ space group is face-centered cubic, allowing the model to be simplified by using a rhombohedral primitive unit cell, which is one-quarter the size of the conventional cubic cell and contains 48 T-sites. In this context, the A $Si_{36}Al_{12}O_{96}H_{12}$ moiety was used to model the Y zeolite with Si/Al molar ratio approximating 3[1]. A Cu^+ cation was stabilized in the 6-membered rings of Y zeolite based on the experimentally observed Cu^+-O-Si species. Moreover, a 13-atom (Pd_9Cu_4) cluster was introduced within the 12-membered channel of Y zeolite and stabilized by two Si-O-Al moieties, which simulate the encapsulated PdCu alloy.

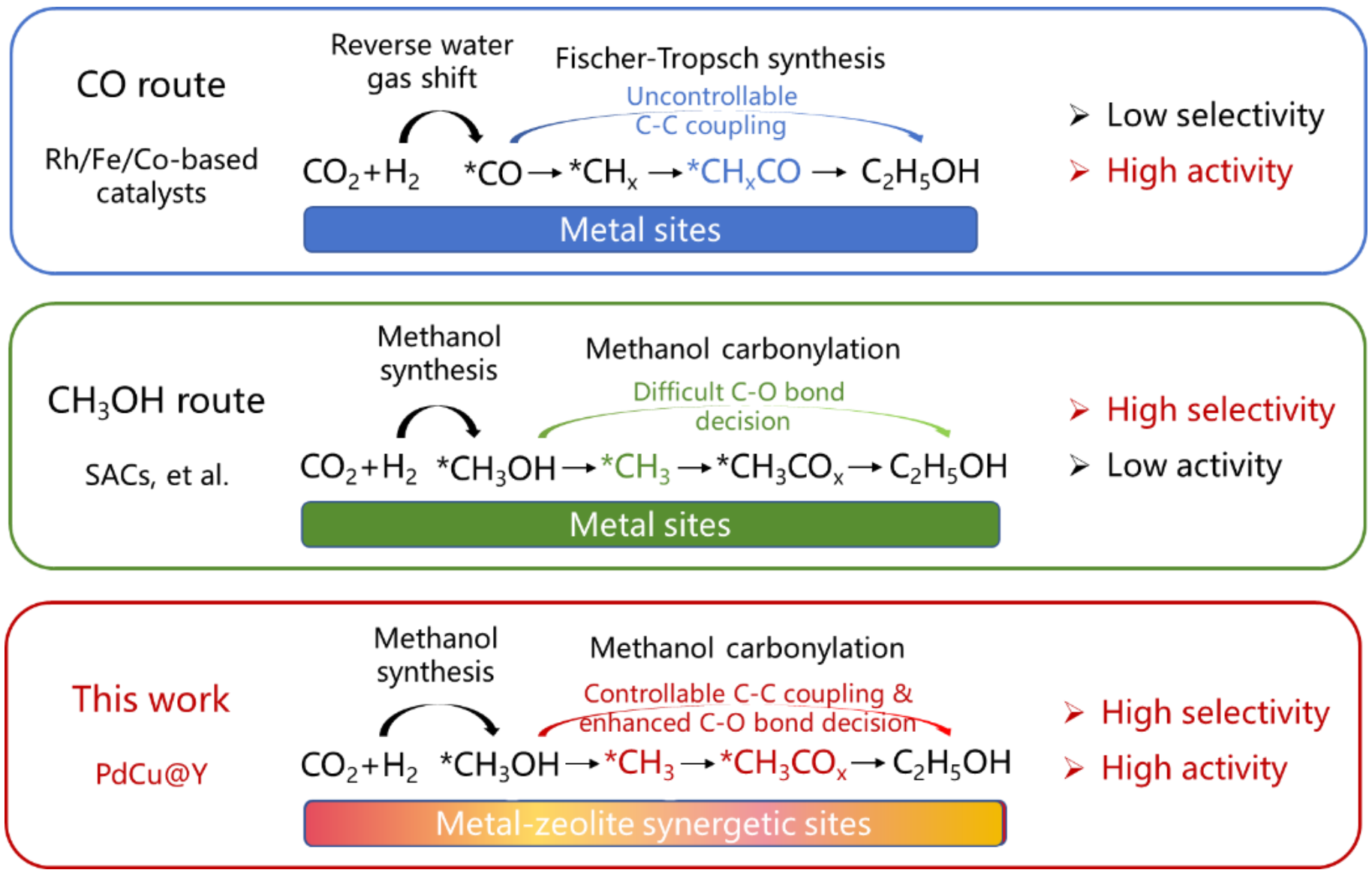

Figure S1. Schematic comparison of metal-zeolite synergetic catalyst and reported metal catalysts for CO_2 hydrogenation to ethanol.

Table S1. Input quantity of metal salt and actual contents of Pd and Cu.

Catalysts	$PdCl_2$ (mg)	$CuCl_2 \cdot 2H_2O$ (mg)	Pd content [a] (wt%)	Cu content [a] (wt%)	Pd/Cu molar ratio
Pd@Y	66.6	0	1.08	/	/
$Pd_{2.3}Cu$@Y	44.4	35.5	0.58	0.15	2.3
$Pd_{1.3}Cu$@Y	33.3	53.3	0.42	0.20	1.3
$Pd_{0.9}Cu$@Y	22.2	71.0	0.31	0.21	0.9
$Pd_{0.3}Cu$@Y	13.3	85.3	0.17	0.40	0.3
Cu@Y	0	106.6	/	0.32	/
$Pd_{1.2}Cu$/Y	14.0	10.7	0.37	0.19	1.2

[a] Determined by ICP-OES.

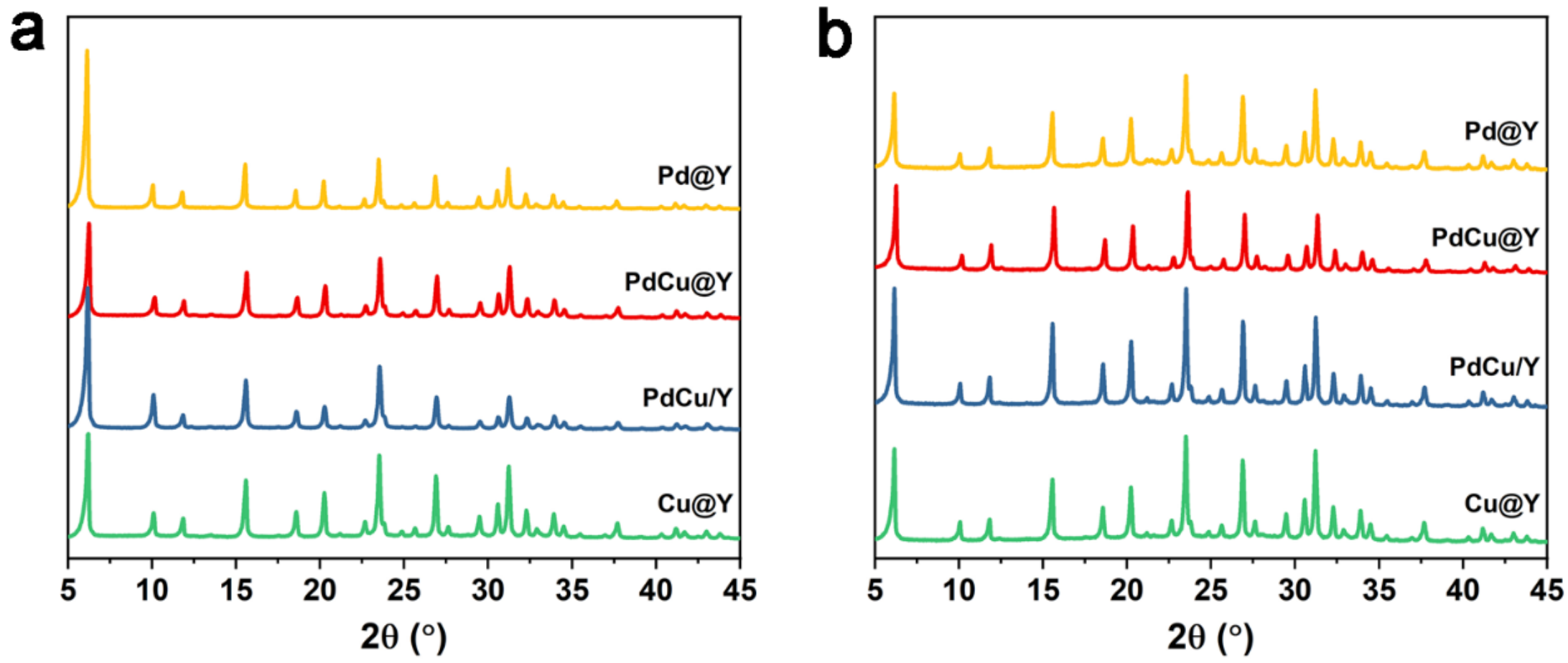

Figure S2. XRD patterns of (a) fresh and (b) spent Pd@Y, PdCu@Y, PdCu/Y and Cu@Y.

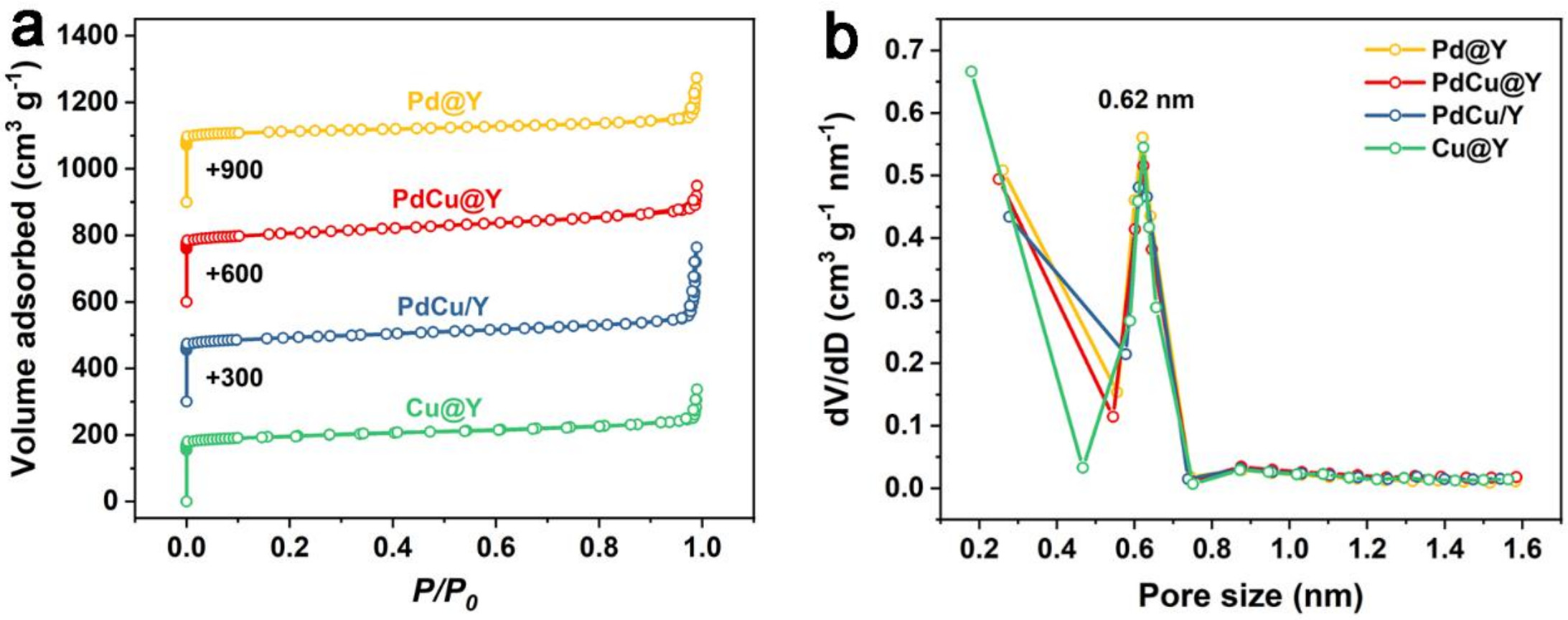

Figure S3. (a) N_2 adsorption-desorption isotherms and (b) micropore size distribution of Pd@Y, PdCu@Y, PdCu/Y and Cu@Y.

Table S2. Properties of Pd@Y, PdCu@Y, PdCu/Y and Cu@Y.

Catalysts	BET surface area [a] (m^2 g^{-1})	Pore volume [a] (cm^3 g^{-1})	Pore diameter [a] (nm)	Si/Al molar ratio [b]
Pd@Y	791	0.58	0.62	3.2
PdCu@Y	758	0.54	0.62	3.1
PdCu/Y	711	0.72	0.62	2.9
Cu@Y	726	0.52	0.62	3.4

[a] Determined by N_2 physisorption.

[b] Determined by EDS.

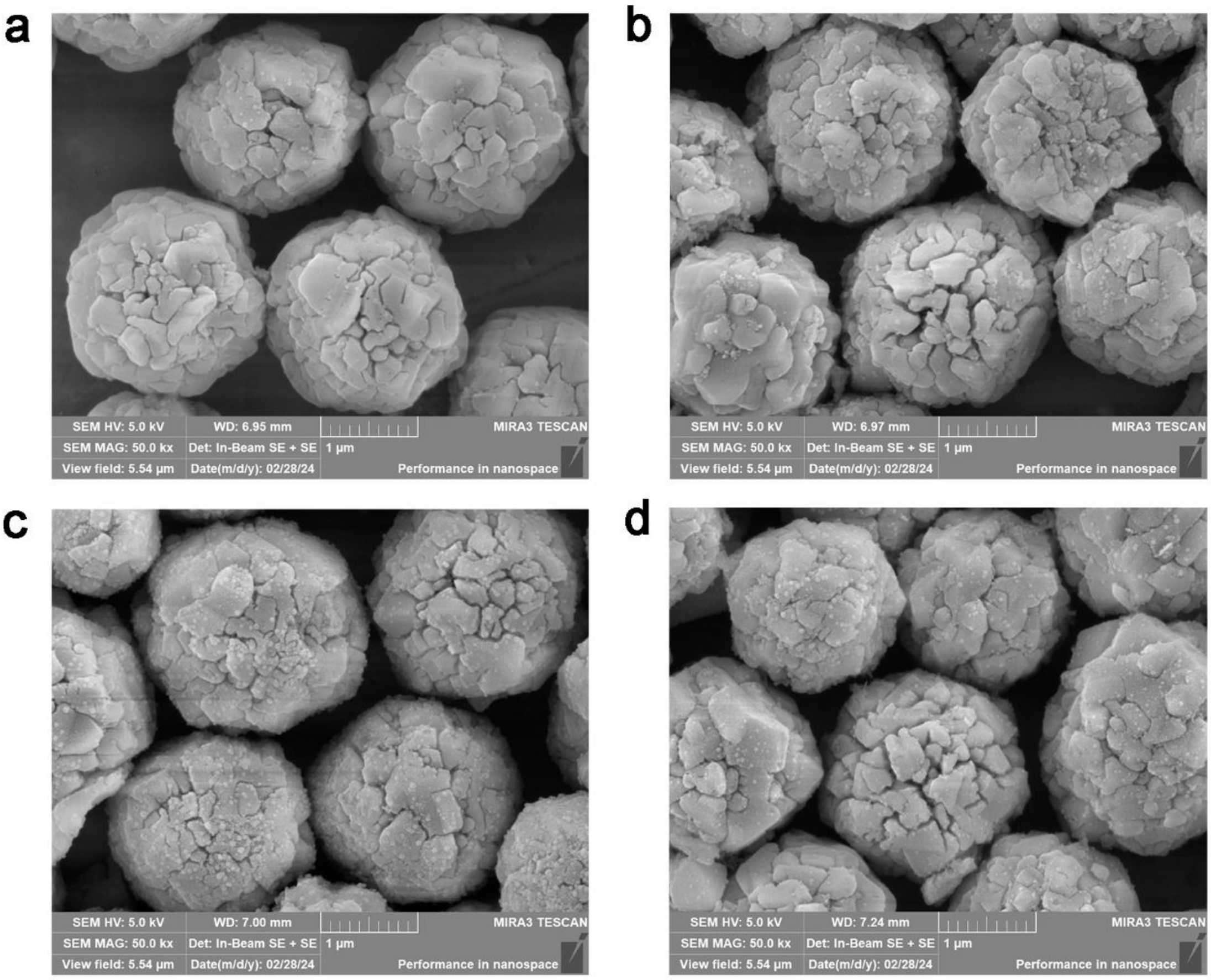

Figure S4. SEM images of (a) Pd@Y, (b) PdCu@Y, (c) PdCu/Y and (d) Cu@Y.

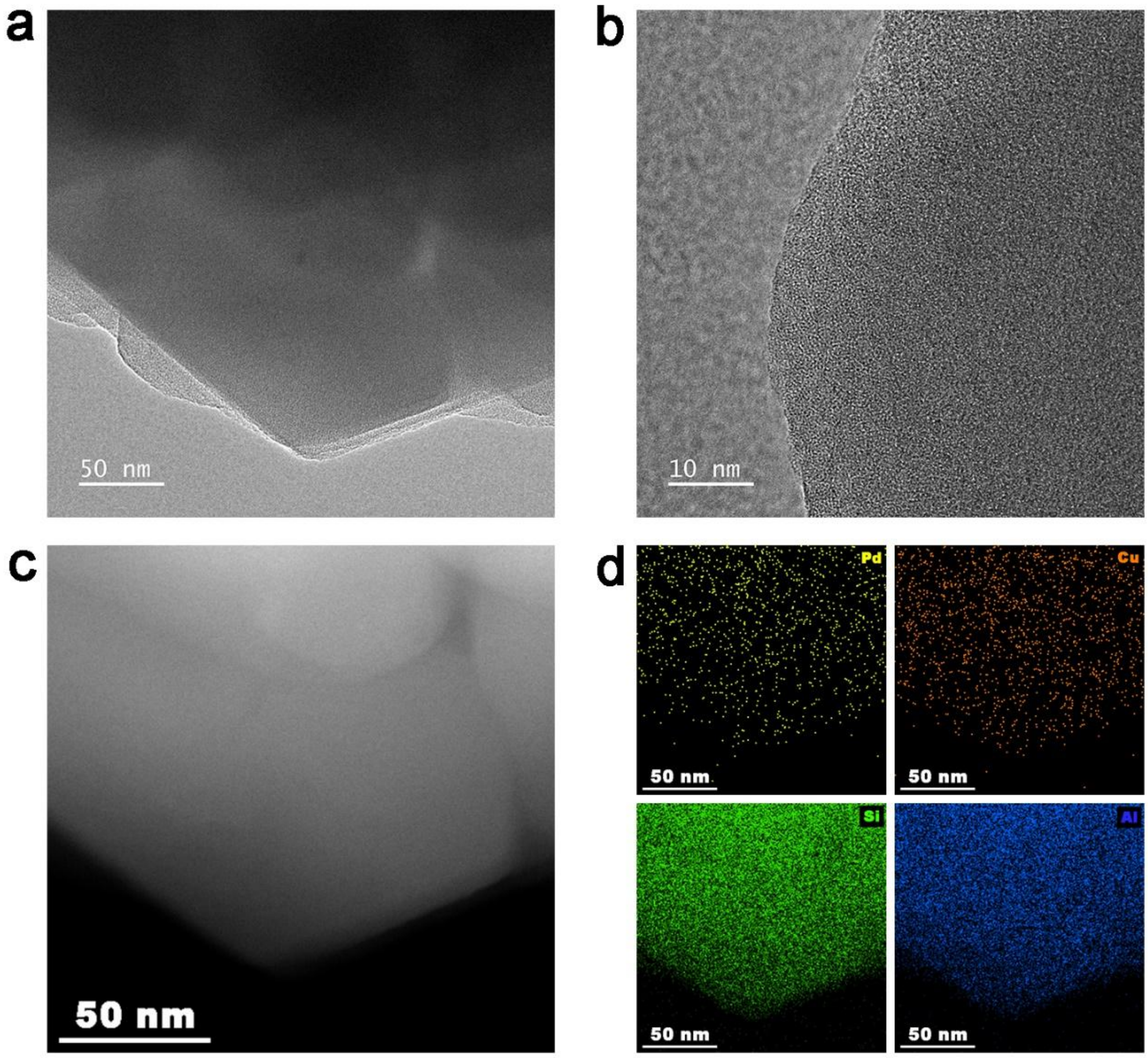

Figure S5. (a) TEM, (b) HR-TEM, (c) HAADF-STEM images and corresponding elementary mapping of fresh PdCu@Y.

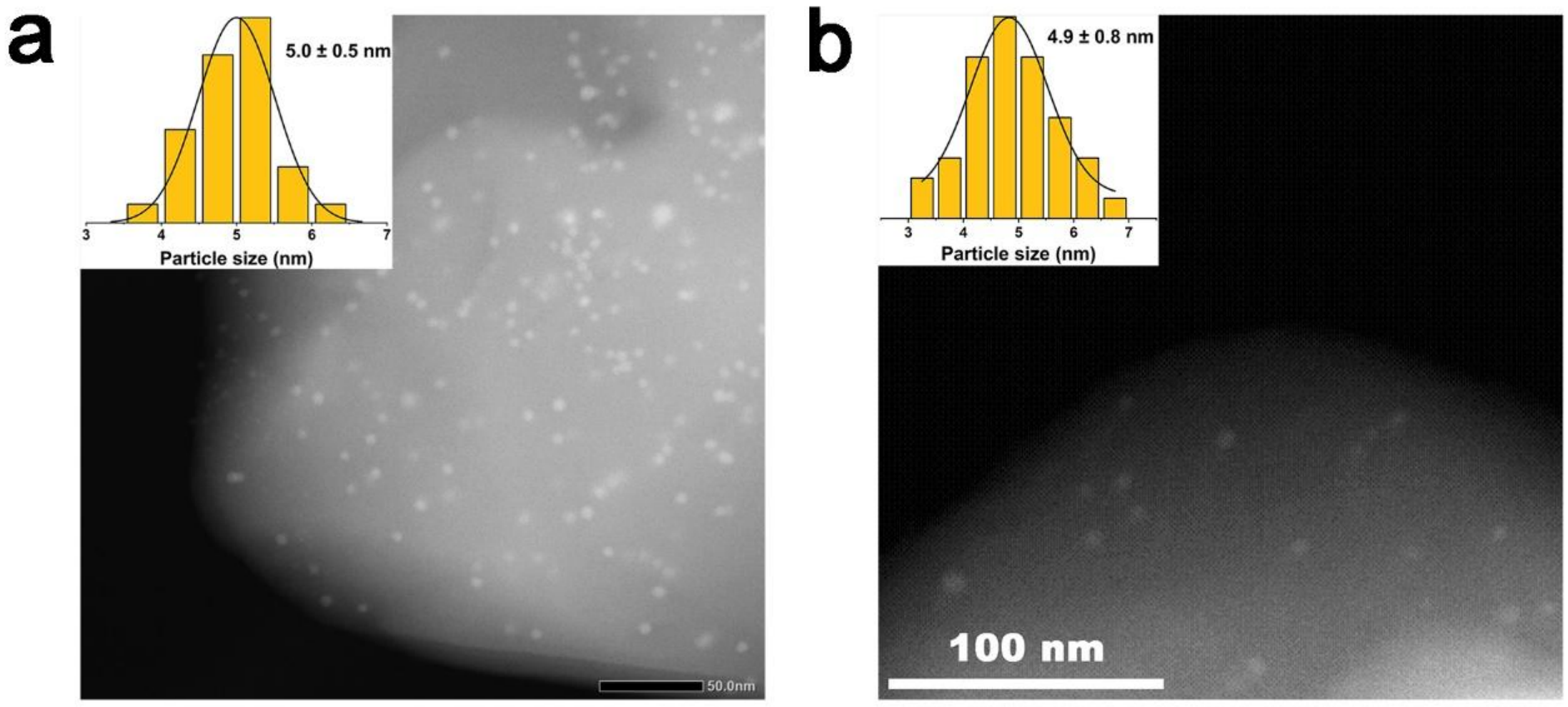

Figure S6. HAADF-STEM and corresponding particle size distribution of (a) reduced PdCu@Y and (b) spent PdCu@Y.

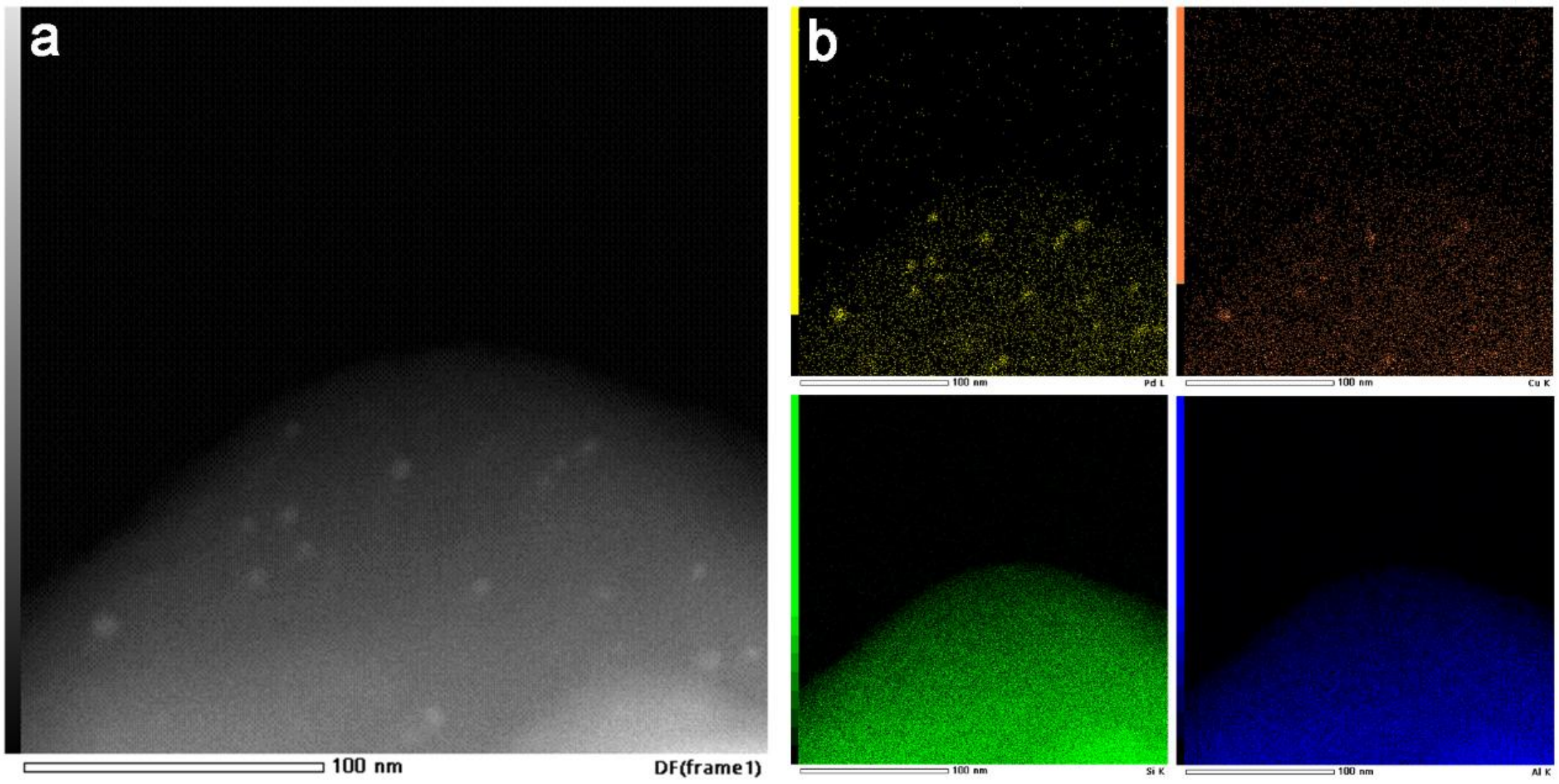

Figure S7. (a) HAADF-STEM and corresponding (b) EDS mapping of reduced PdCu@Y.

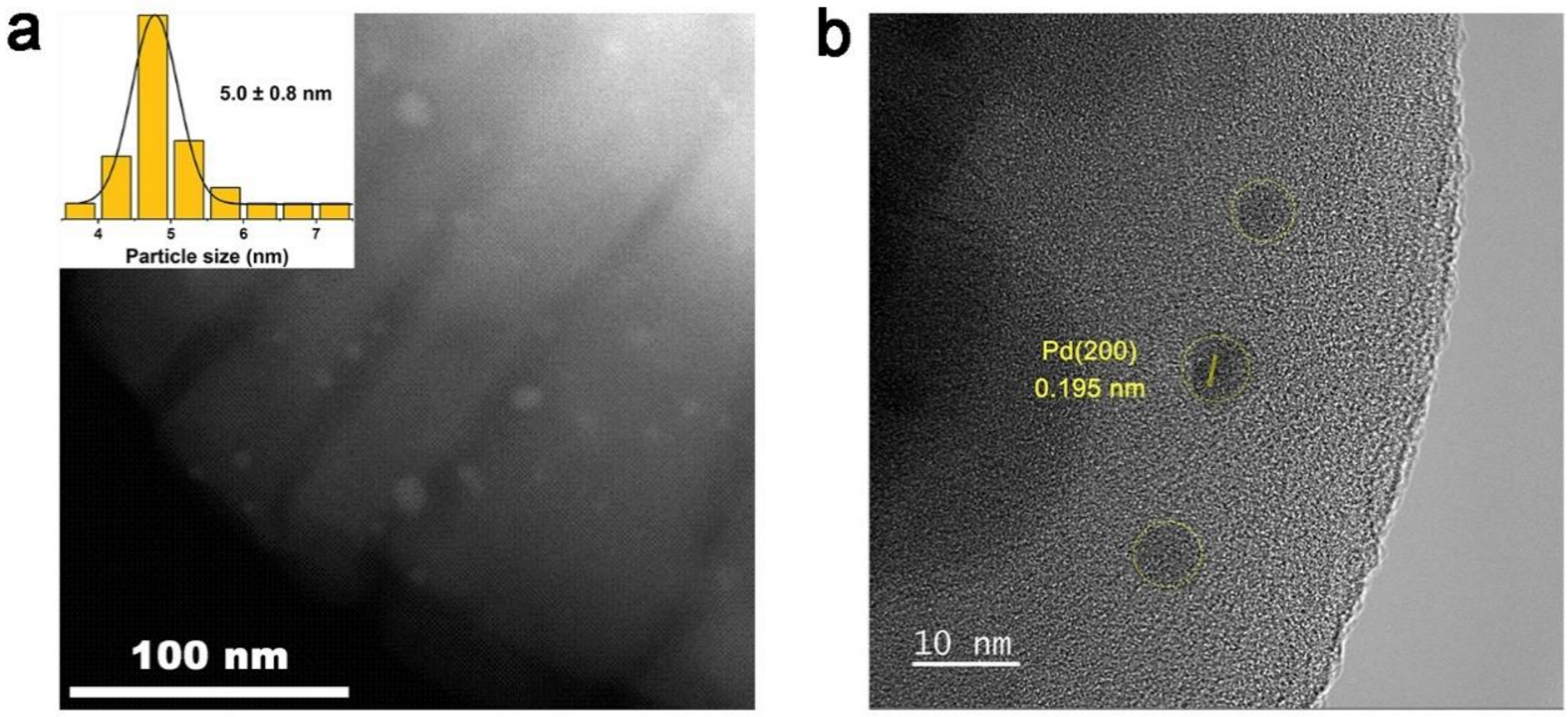

Figure S8. (a) HAADF-STEM and corresponding particle size distribution, and (b) HR-TEM image of reduced Pd@Y.

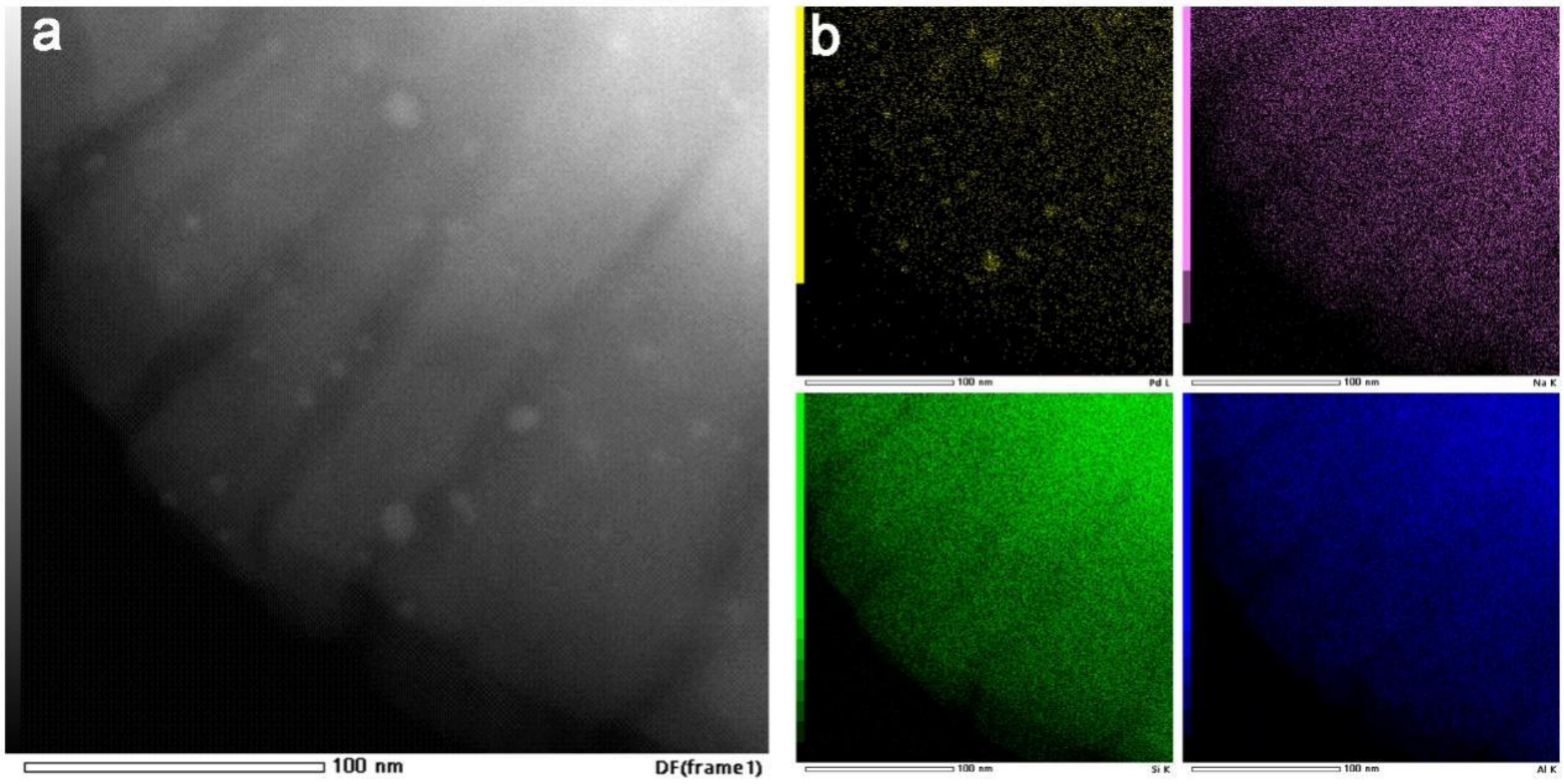

Figure S9. (a) HAADF-STEM and corresponding (b) EDS mapping of reduced Pd@Y.

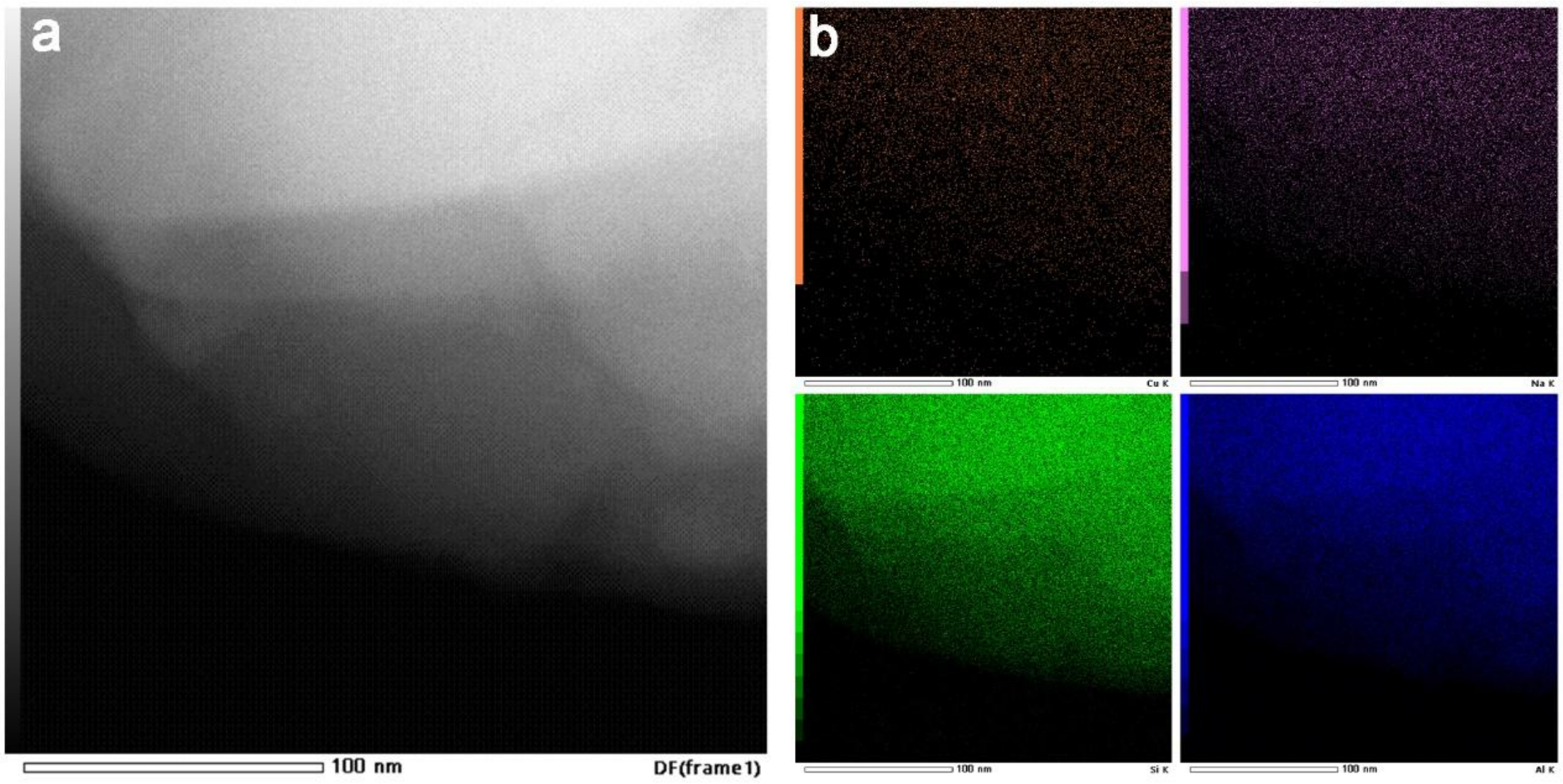

Figure S10. (a) HAADF-STEM and corresponding (b) EDS mapping of reduced Cu@Y.

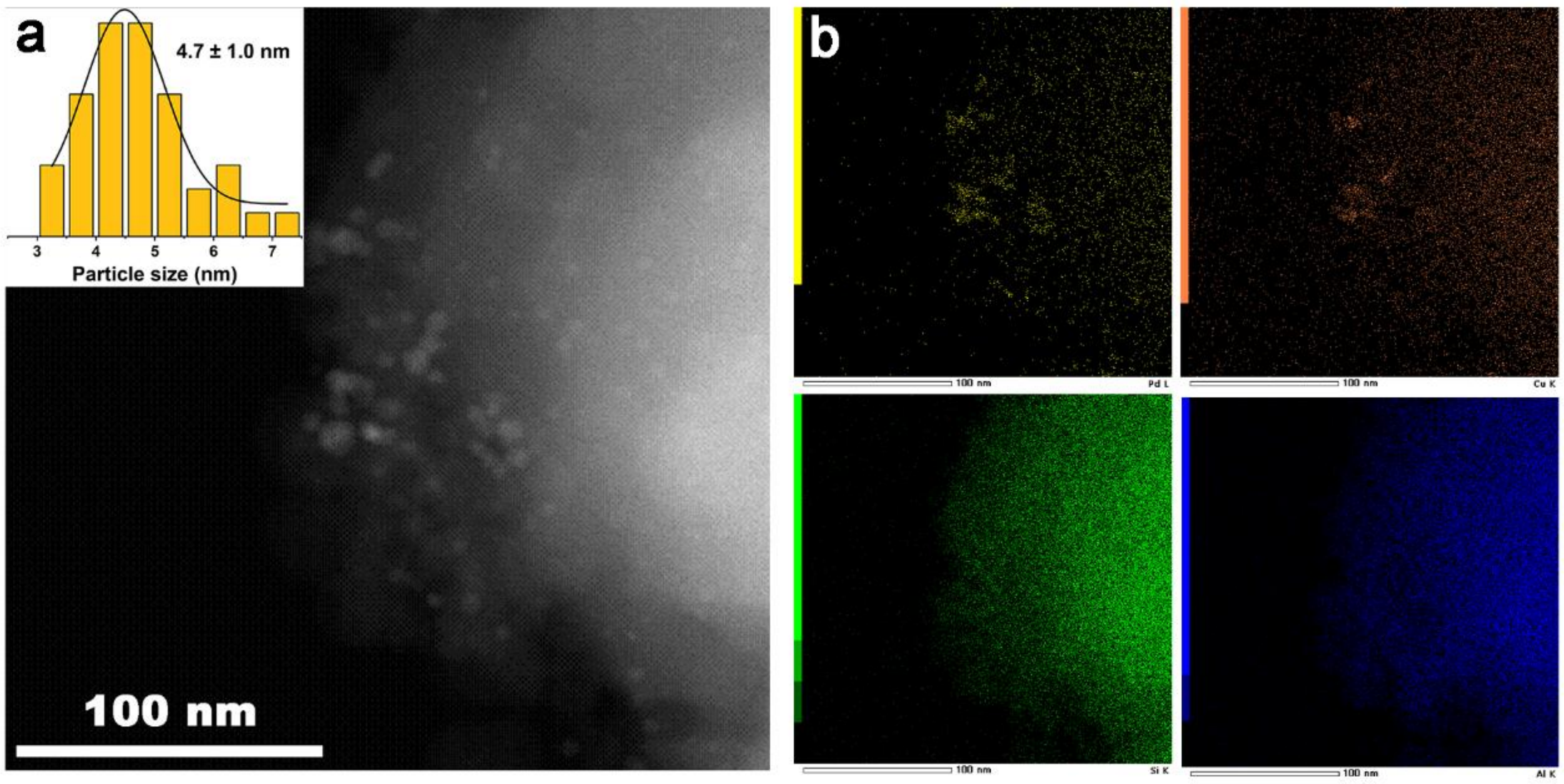

Figure S11. (a) HAADF-STEM and corresponding particle size distribution, and (b) EDS mapping of reduced PdCu/Y.

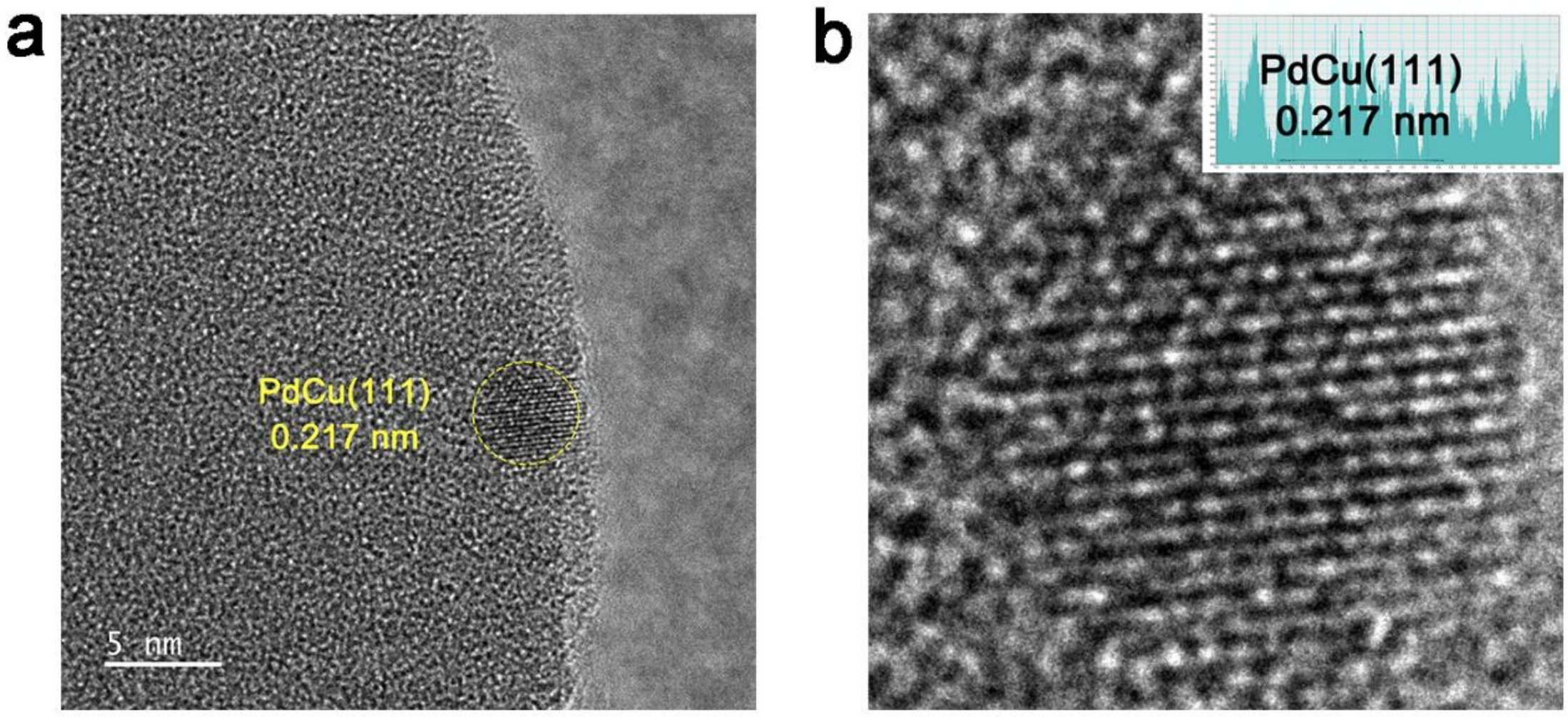

Figure S12. (a) HR-TEM and (b) corresponding lattice fringe of spent PdCu@Y.

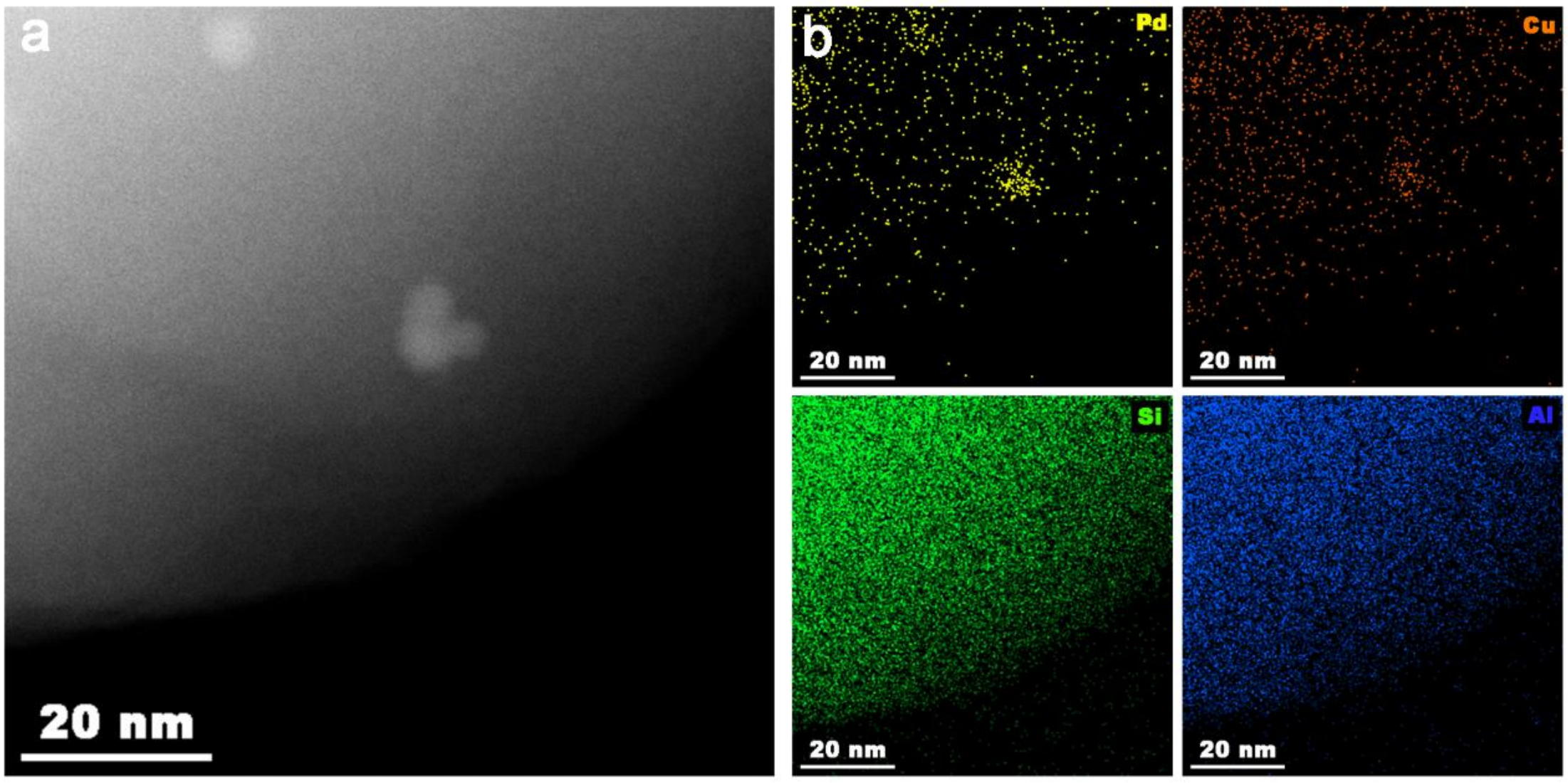

Figure S13. (a) HAADF-STEM and corresponding (b) EDS mapping of spent PdCu@Y.

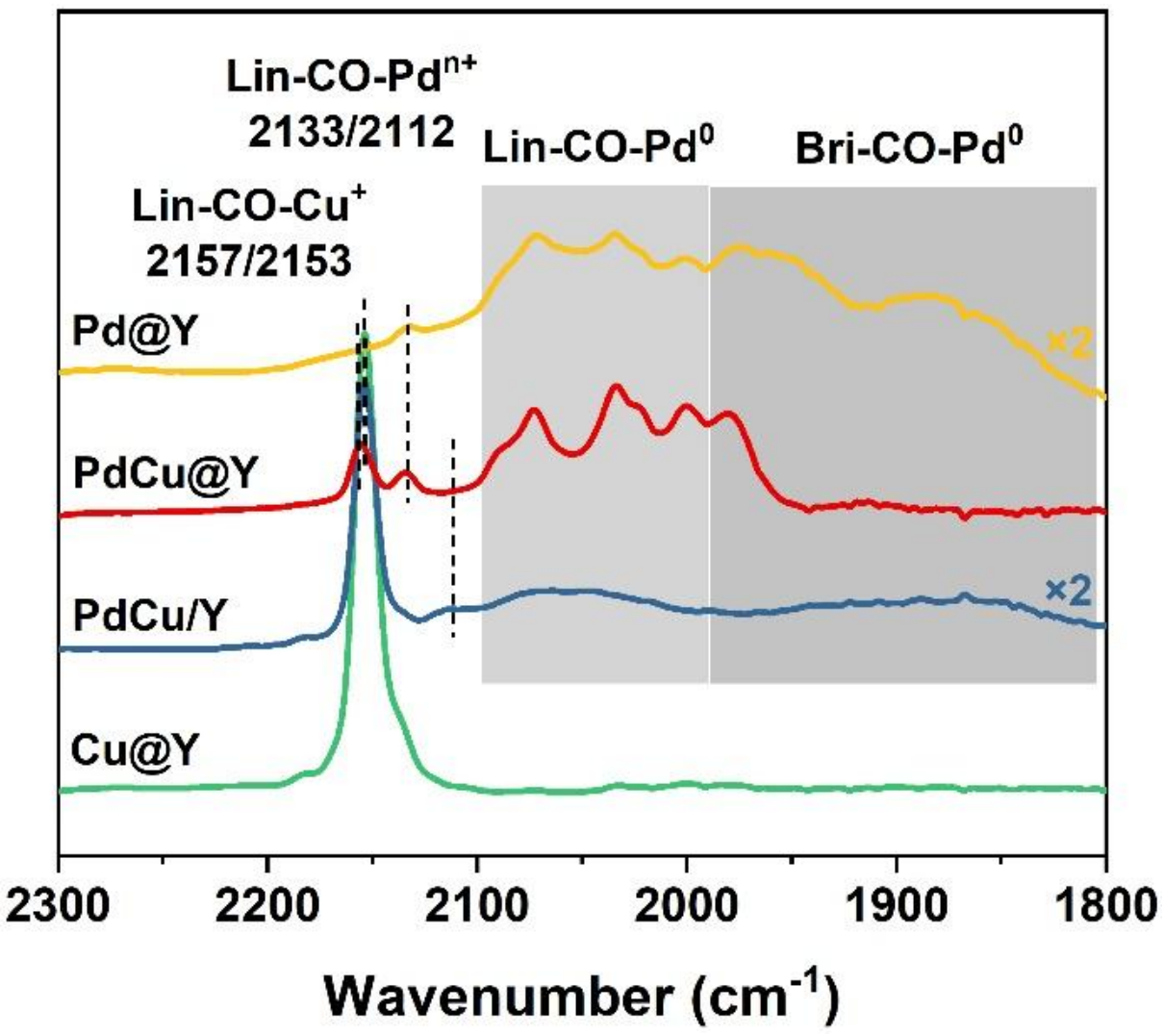

Figure S14. CO-DRIFTS of reduced Pd@Y, PdCu@Y, PdCu/Y and Cu@Y after Ar purge for 30 minutes.

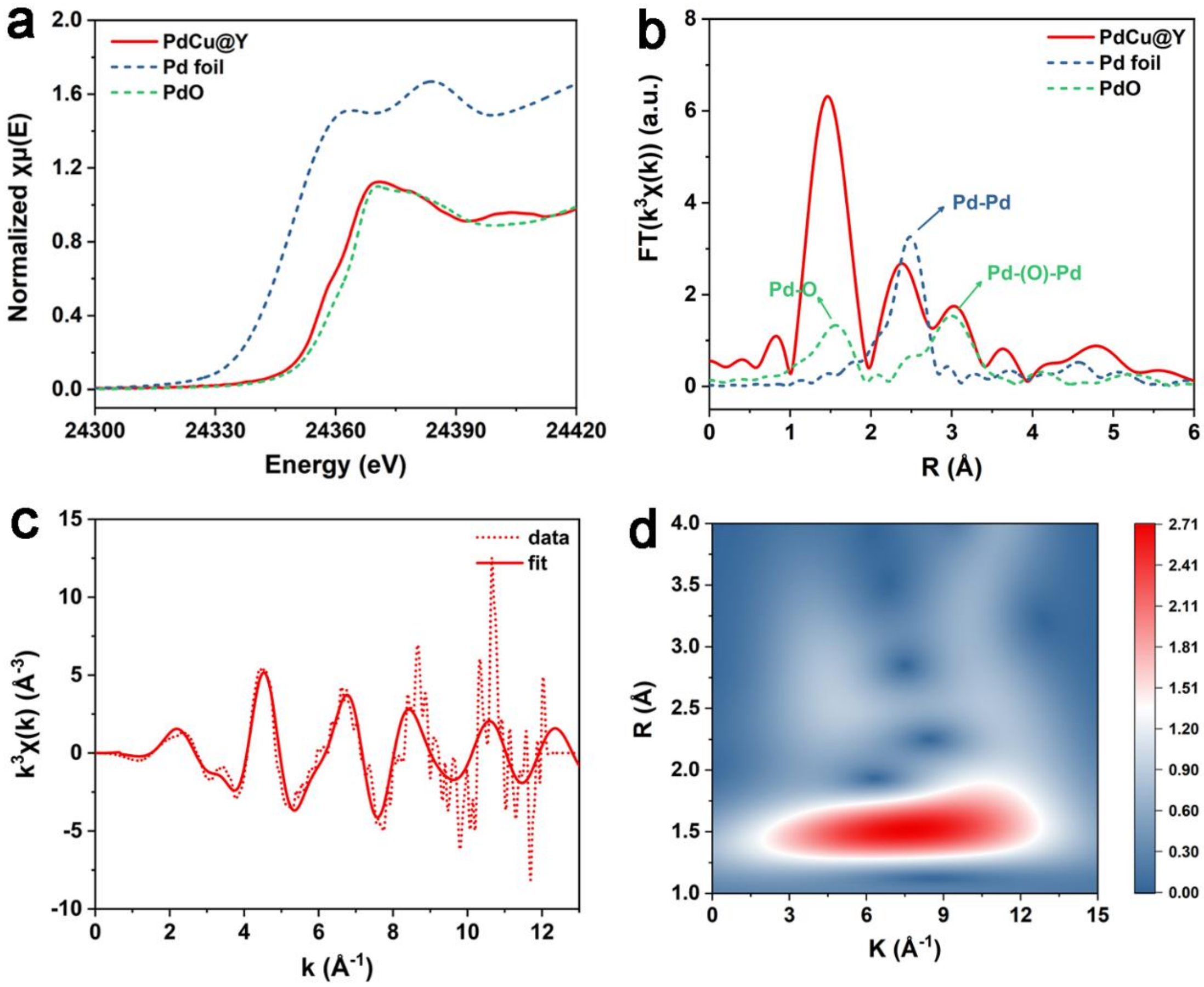

Figure S15. XAFS characterization of fresh PdCu@Y. (a) Pd K-edge XANES spectra. (b) Fourier transform of k^3-weighted EXAFS spectra. (c) EXAFS oscillations of k^3-weighted EXAFS spectra. (d) Wavelet transform of EXAFS spectra at Pd K-edge.

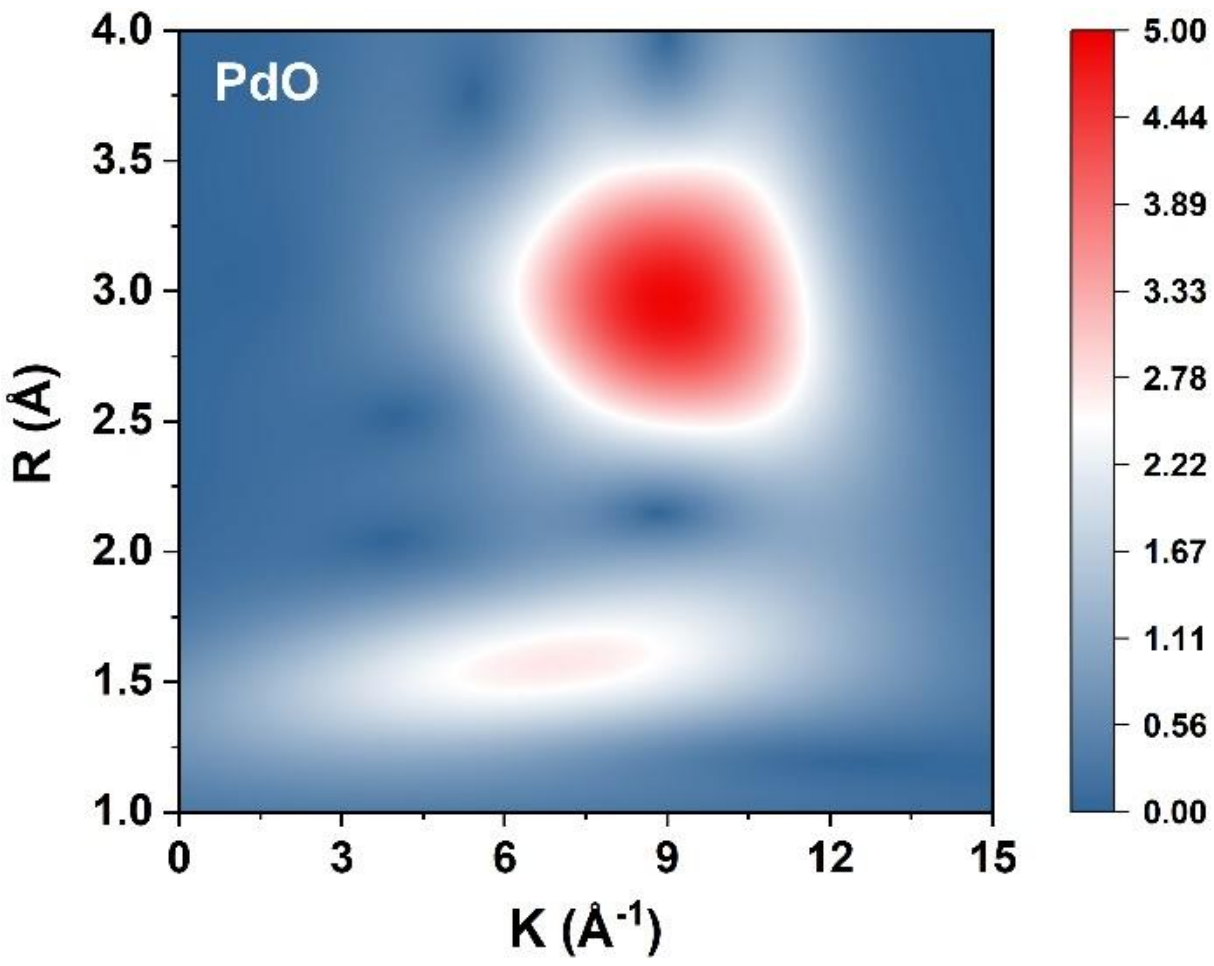

Figure S16. Wavelet transform of EXAFS spectra at Pd K-edge of PdO.

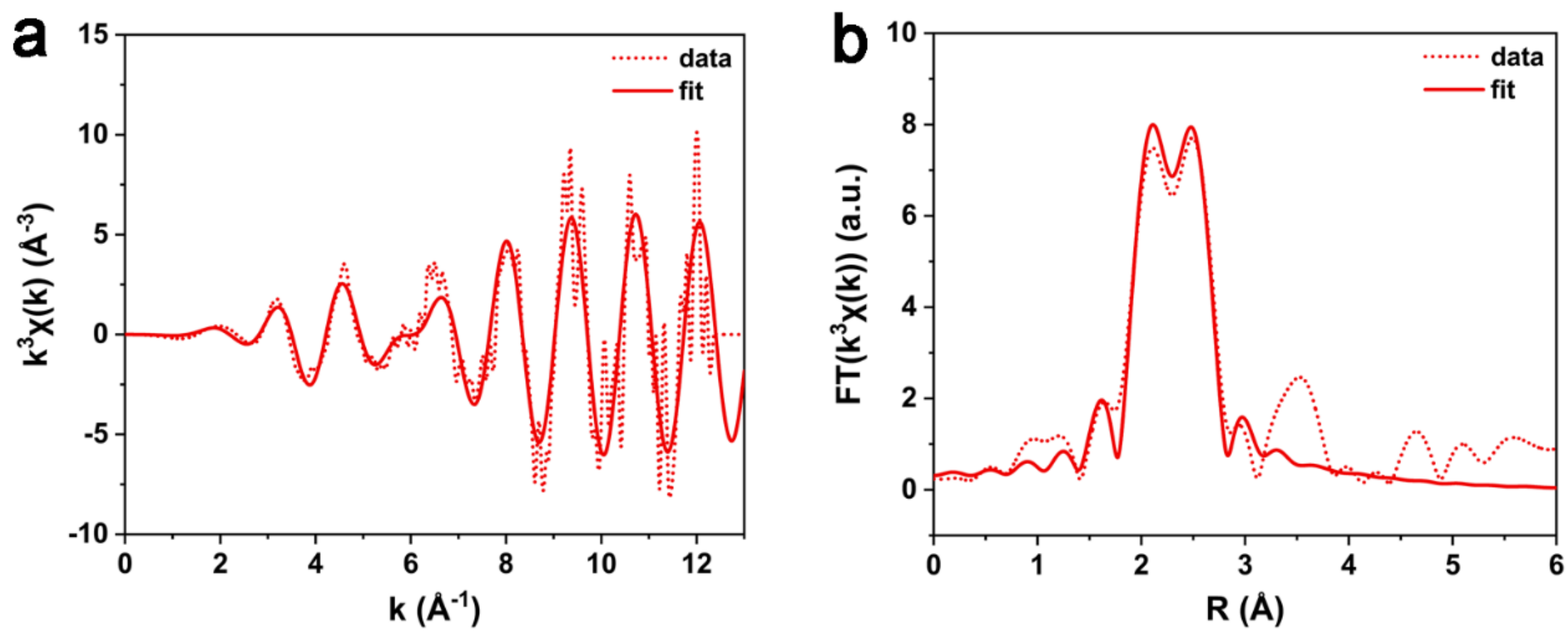

Figure S17. (a) EXAFS oscillations and (b) Fourier transform of k^3-weighted EXAFS spectra of reduced PdCu@Y.

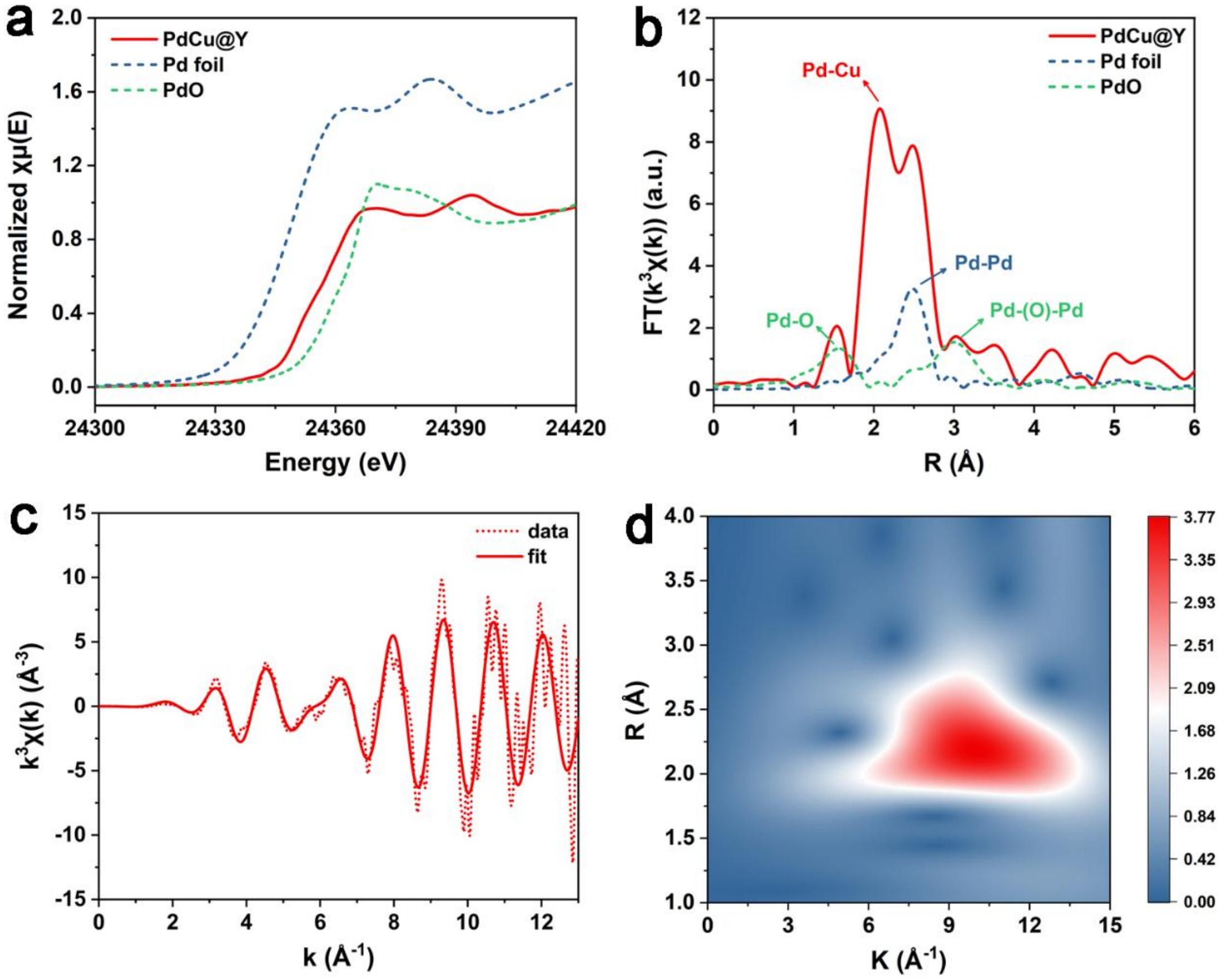

Figure S18. XAFS characterization of spent PdCu@Y. (a) Pd K-edge XANES spectra. (b) Fourier transform of k^3-weighted EXAFS spectra. (c) EXAFS oscillations of k^3-weighted EXAFS spectra. (d) Wavelet transform of EXAFS spectra at Pd K-edge.

Table S3. Structural parameters at the Pd K-edge extracted from quantitative EXAFS curve fittings using either Pd-Cu or Pd-Pd scattering path of PdCu@Y.

Sample	Shell	C.N.	R	ΔE_0	R-factor
fresh	Pd-O	3.16±0.74	2.01±0.05	1.68±1.31	0.044
	Pd-Pd	5.98±1.55	2.69±0.08		
reduced	Pd-Cu	1.57±0.20	2.59±0.06	-2.51±0.91	0.010
	Pd-Pd	5.07±0.86	2.66±0.05		
spent	Pd-Cu	2.42±0.78	2.60±0.02	-3.33±1.32	0.015
	Pd-Pd	5.30±0.79	2.67±0.10		

C.N., coordination number; R, bonding distance; ΔE_0, inner potential shift; R-factor, the goodness of the fit.

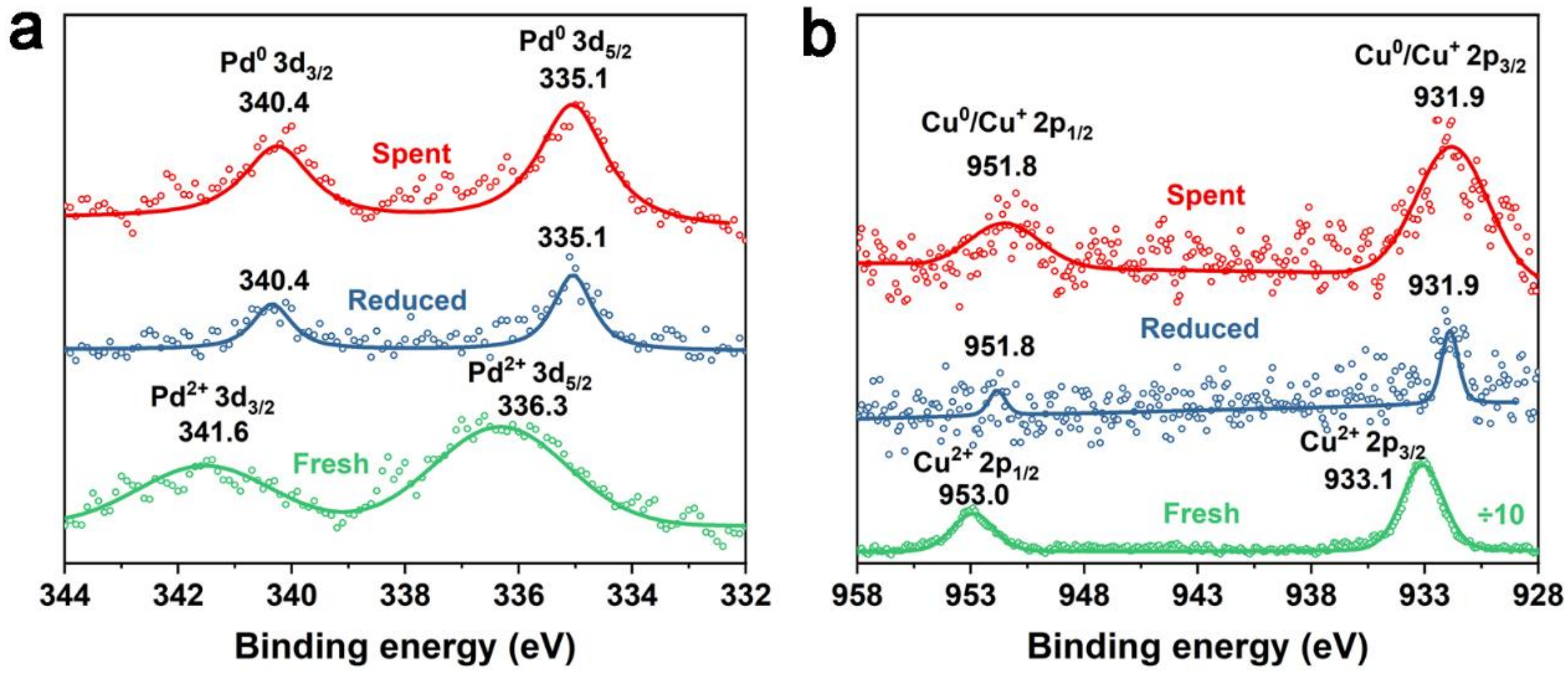

Figure S19. XPS patterns of (a) Pd 3d and (b) Cu 2p of PdCu@Y.

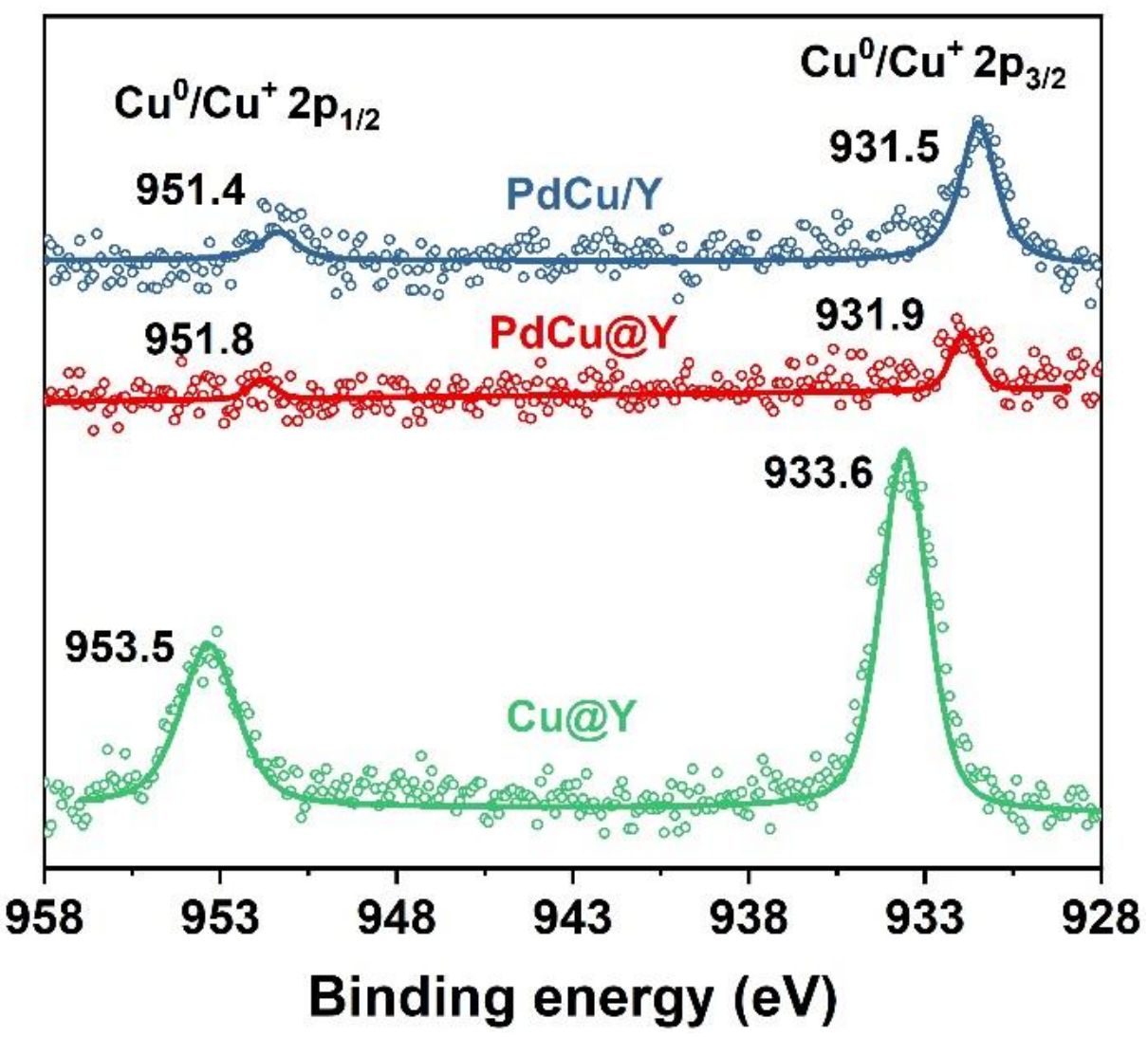

Figure S20. XPS patterns of Cu 2p of reduced PdCu@Y, PdCu/Y and Cu@Y.

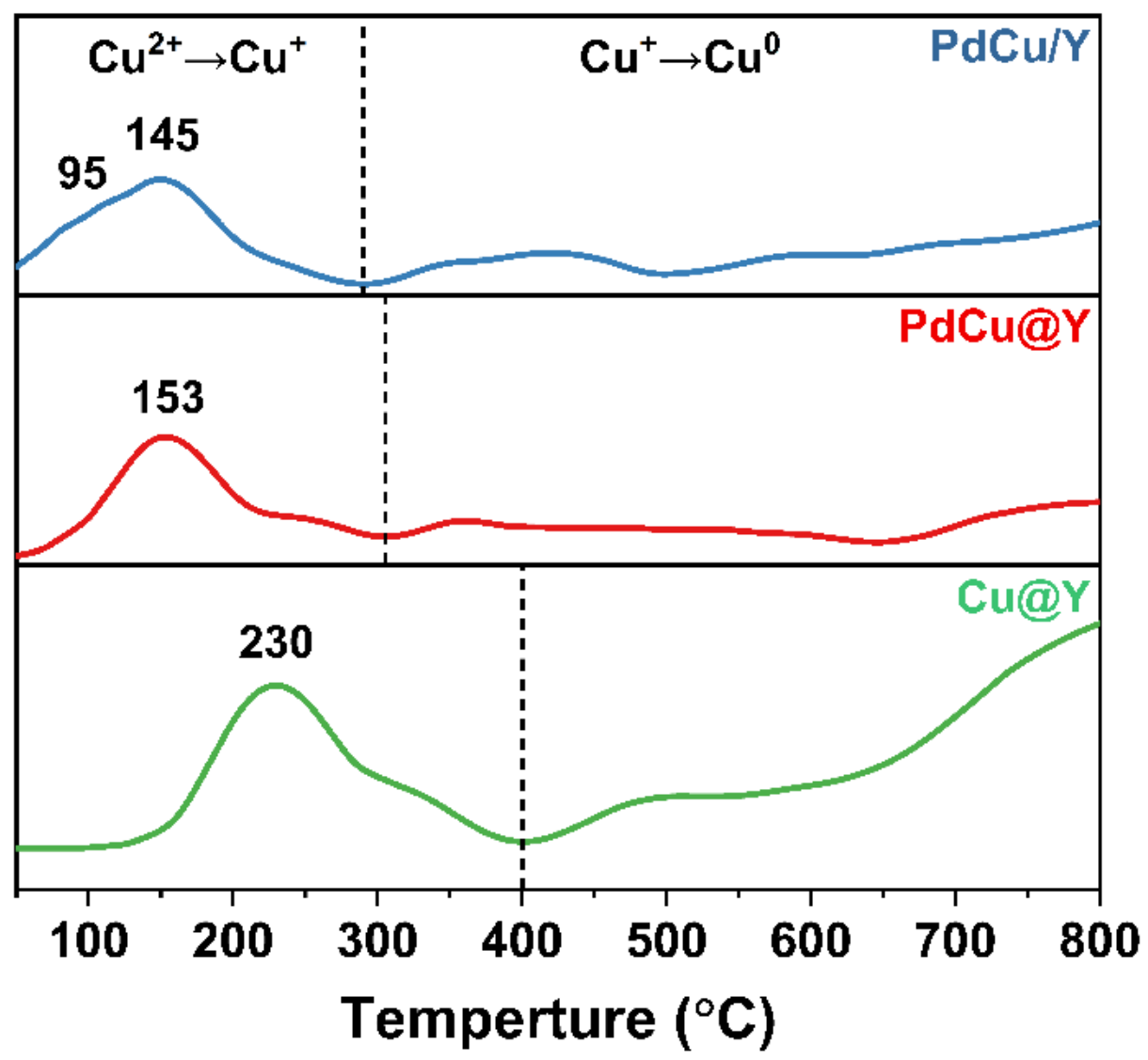

Figure S21. H_2-TPR of PdCu@Y, PdCu/Y and Cu@Y.

As for Cu@Y (Figure S21), the H_2 reduction peaks at 230 ºC and above 400 ºC were assigned to the reductions of Cu^{2+} to Cu^{+} and Cu^{+} to Cu^{0}, respectively[2,3]. Therefore, Cu^{+} dominated in the Cu@Y catalyst after reduction treatment. As the introduction of Pd, all reduction peaks obviously shifted to low-temperature regions, suggesting that the reduction of Cu^{2+} was promoted due to the hydrogen spillover of Pd[4]. Noted that PdCu@Y showed higher reduction temperatures compared with PdCu/Y, which can be illustrated by the stronger metal-zeolite interactions on encaged sample.

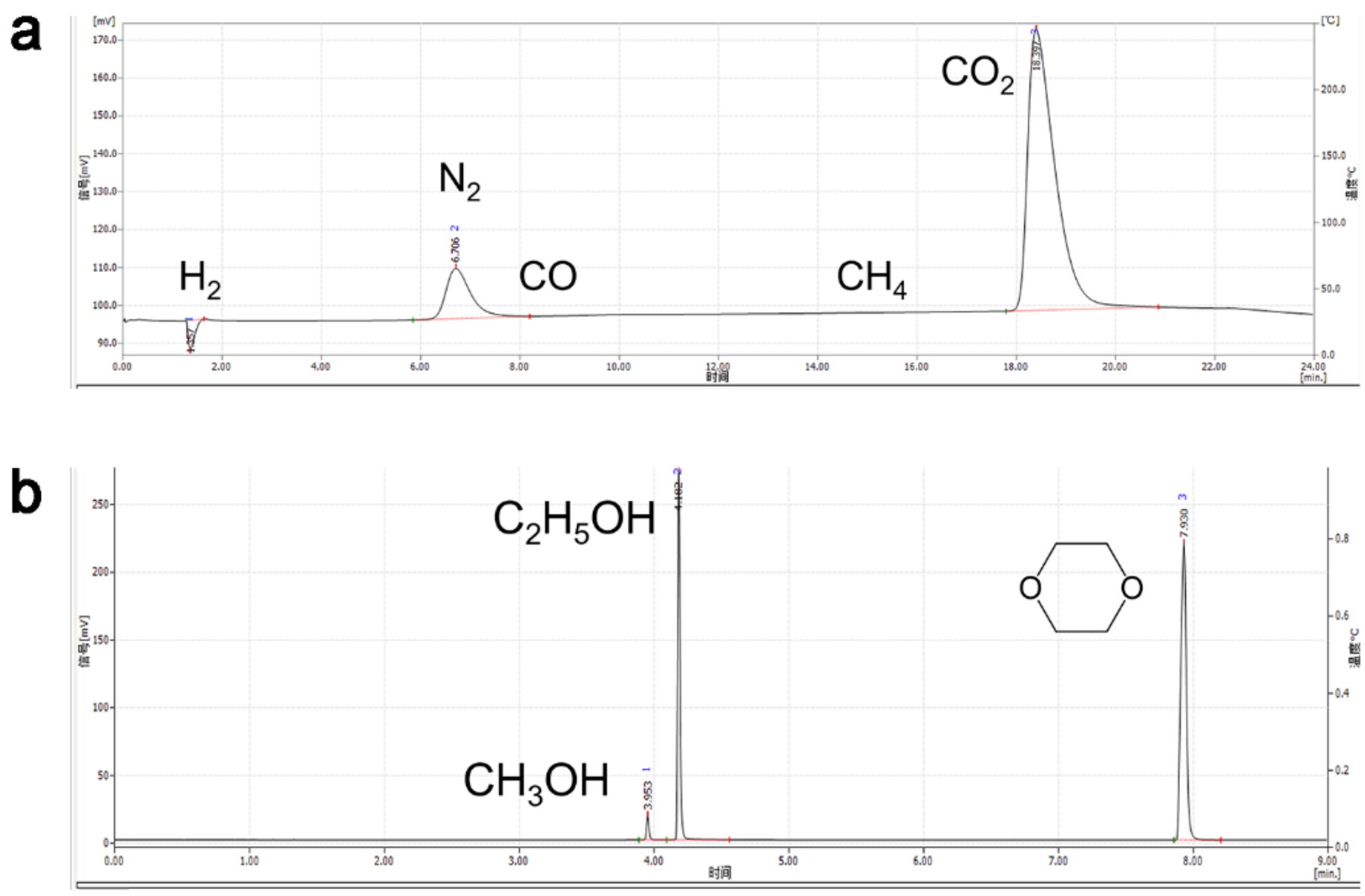

Figure S22. Gas chromatography results of (a) gaseous and (b) liquid products on PdCu@Y in CO_2 hydrogenation. Dioxane was added into liquid products as internal standard.

Table S4. CO_2 hydrogenation to ethanol performance

Catalysts	CO_2 conversion (%)	Ethanol selectivity (%)	Methanol selectivity (%)	Ethanol yield (mg)	Ethanol STY (mmol g_{cat}^{-1} h^{-1})	Ethanol STY (mg g_{cat}^{-1} h^{-1})	Ethanol STY (mmol g_M^{-1} h^{-1})
Pd@Y	0.64	62.4	37.6	3.2	0.22	10.1	20.2
$Pd_{2.3}Cu$@Y	0.95	95.9	4.1	6.9	0.50	23.0	68.1
$Pd_{1.3}Cu$@Y	8.98	93.2	6.8	63.0	4.55	209.3	734.2
$Pd_{0.9}Cu$@Y	6.32	70.2	29.8	33.6	2.42	111.3	464.9
$Pd_{0.3}Cu$@Y	2.35	13.9	86.1	2.3	0.18	8.3	31.0
Cu@Y	0.13	0	100	0	0	0	0
$Pd_{1.2}Cu$/Y	0.09	7.9	92.1	0.05	0.004	0.2	0.7

Reaction conditions: 50 mg catalyst, 8 mL H_2O, H_2/CO_2 = 3, 4 MPa, 200 ºC, 6 h.

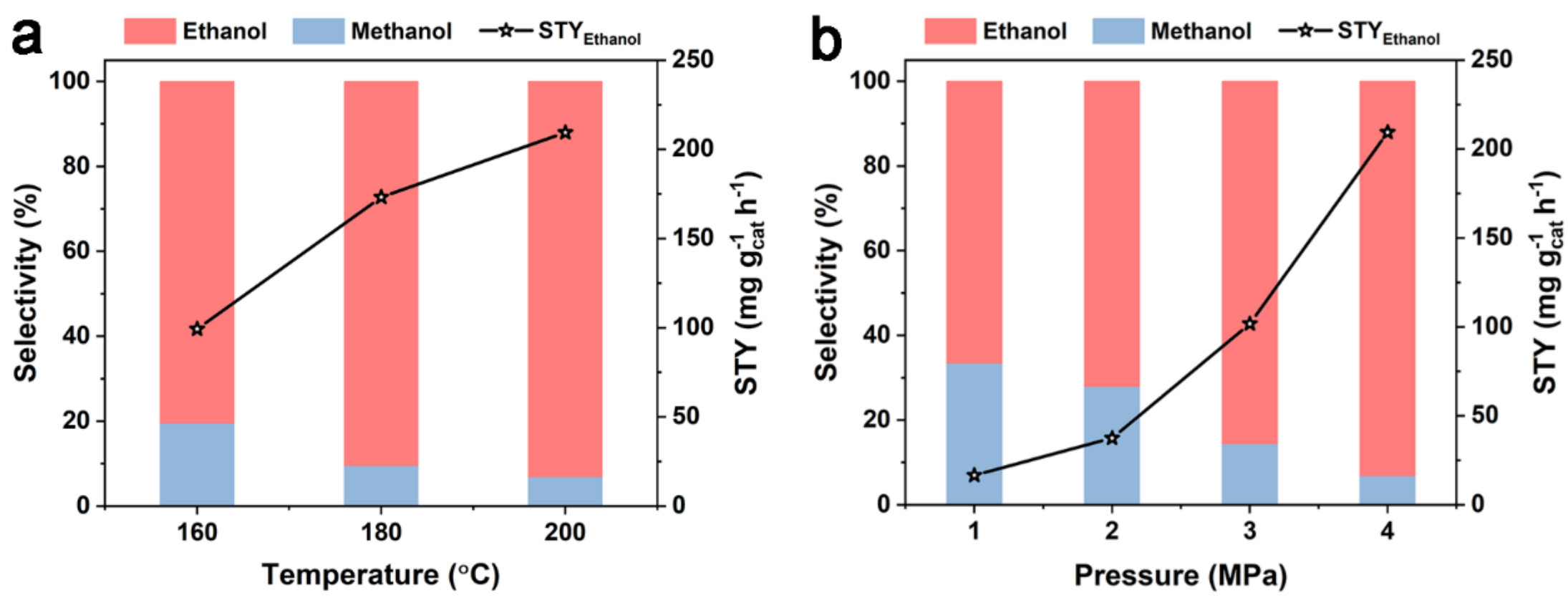

Figure S23. CO_2 hydrogenation performance of PdCu@Y at different (a) temperatures and (b) pressures.

Table S5. Catalytic performance comparison of CO_2 hydrogenation to ethanol.

Catalysts	Reactor	T (ºC)	P (MPa)	H_2/CO_2	WHSV (L g_{cat}^{-1} h^{-1})	*t* (h)	Ethanol selectivity (%)	Ethanol STY (mg g_{cat}^{-1} h^{-1})	Ref.
Na-ZnFe@C	Fix-bed	320	5	3	9	/	18.8	158.1	5
5La-CuFeO$_x$	Fix-bed	320	3	3	3	/	33.8	99.4	6
KFeCuZn/ZrO$_2$	Fix-bed	320	4	3	12	/	14.6	161.0	7
MnCuK-FeC(1)/ CuZnAlZr(1)	Fix-bed	300	3	3	6	/	9.3	115.8	8
FeCuGaZn-0.75	Fix-bed	340	5	3	6	/	88.9	88.9	9
Cu/UIO-67	Fix-bed	260	0.1	3	24	/	1	0.7	10
5Co/S-1	Fix-bed	250	2	3	4.67	/	27	37.7	11
Co20MgAl	Fix-bed	300	4	3	2	/	10.6	42.2	12
$Co_{1.8}Cu_{0.9}AlO_x$	Fix-bed	250	3	3	10.625	/	17.3	109.9	13
600-CDM	Fix-bed	270	3	3	3	/	16.9	11.4	14
3K-CuCo-1	Fix-bed	260	3	3	6	/	21	104.0	15
Rh/TiO$_2$	Fix-bed	260	3	3	6	/	1.4	5.5	16
Na-Rh-FeO$_x$/ZSM5	Fix-bed	200	1.5	3	3.6	/	11	1.2	17
1%RhFeK/TiO$_2$-DP	Fix-bed	200	3	3	2	/	33.4	186.8	18
2Rh0.5Fe0.5Na/CeO$_2$	Fix-bed	250	3	3	6	/	29.8	58.4	19
1Pd$_2$Ce@Si$_{16}$	Fix-bed	250	3	3	3	/	98.7	39.4	20
0.19Na-Rh@S-1	Fix-bed	250	5	3	6	/	24	24.8	21
0.1Pd/Fe$_3$O$_4$	Fix-bed	300	0.1	3	/	/	97.4	19.0	22
Na–CuCo-9	Fix-bed	330	4	1	2	/	8	35.7	23
$CoGa_{1.0}Al_{1.0}O_4/SiO_2$	Fix-bed	270	3	3	3	/	20.1	13.8	24
Ru/In$_2$O$_3$-ZrO$_2$	Fix-bed	225	0.6	3	3.6	/	70	0.7	25
IrMo-SiO$_2$	Fix-bed	200	4.9	2	2	/	4.3	1.8	26
Li-RhY	Fix-bed	250	3	3	6	/	2.7	5.5	27
RhLi-SiO$_2$	Fix-bed	240	5	3	6	/	15.5	16.6	28
RhFe-SiO$_2$	Fix-bed	260	5	3	6	/	16	65.8	29
RhFe-TiO$_2$	Fix-bed	270	2	1	8	/	6.4	23.9	30
RhFeLi-TiO$_2$	Fix-bed	250	3	3	6	/	31.3	75.9	31
RhV-MCM41	Fix-bed	250	3	3	6	/	24.1	45.1	32
Cu@Na-Beta	Fix-bed	300	2.1	3	12	/	79	397.9	33
Ir$_1$-In$_2$O$_3$	Tank	200	6	5	/	5	99	45.5	34
Ir$_1$-P$_x$/In$_2$O$_3$	Tank	180	1	3	/	8	98.5	153.2	35
RhFeO$_x$/P25	Tank	220	3	3	/	5	87.8	432.4	36
Rh$_1$/CeTiO$_x$	Tank	250	3	3	/	5	99.1	0.3	37
Rh/CNP	Tank	250	3	3	/	5	81.8	0.2	38
Pt/Co$_3$O$_4$	Tank	200	8	3	/	15	47	13.3	39
Au/TiO$_2$	Tank	200	6	3	/	10	99	130.1	40
Cu@m-Beta	Tank	200	4	3	/	5	68	18.0	41
CuCo/MCM-41	Tank	200	4	3	/	/	85.3	0.5	42
1-Pt-Co$_3$O$_4$-250	Tank	240	3.2	3	/	/	87.9	12.2	43

$CoAlO_x$	Tank	200	4	3	/	15	92.1	20.4	44
$CoNiAlO_x$	Tank	200	4	3	/	12	85.7	60.6	45
**PdCu@Y**	**Tank**	**200**	**4**	**3**	**/**	**6**	**93.2**	**209.4**	**This work**

Table S6. Chemisorption and acid amounts of series samples.

Catalysts	H_2 peak area [a] (a.u.)	H_2O peak area [a] (a.u.)	Brønsted acid amount [b] (umol g^{-1})	Lewis acid amount [b] (umol g^{-1})	B/L ratio
Pd@Y	2.71E-08	7.56E-06	75.1	358.6	0.21
PdCu@Y	1.95E-08	3.44E-06	55.0	284.8	0.19
PdCu/Y	8.54E-08	1.23E-06	31.0	246.7	0.13
Cu@Y	1.36E-08	2.84E-06	47.8	488.3	0.10

[a] Determined by H_2-TPD-MS.

[b] Determined by pyridine adsorption.

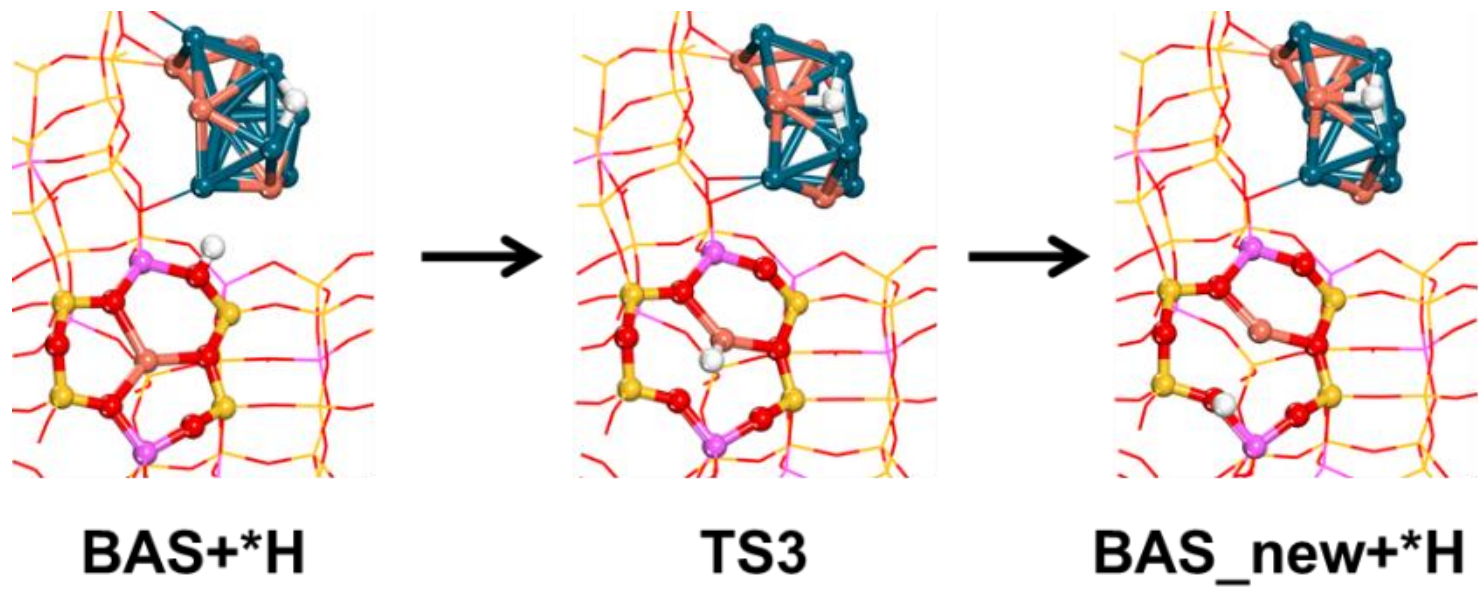

Figure S24. Structural models for BAS formation on PdCu@Y catalyst. Pd (blue), Cu (orange), Si (yellow), Al (pink), O (red), H (white).

Table S7. Assignments of DRIFTS adsorption peaks.

Adsorbed species	Wavenumbers (cm^{-1})	References
$*CO_3^{2-}$	1694	18,35
*HCOO	3032/2977/2818/2719/1618	18,35
$*CH_3O$	2988/2904	18,35
$*CH_3COO$	3032/1730	46,47
$*H_2O$	1646	46,47

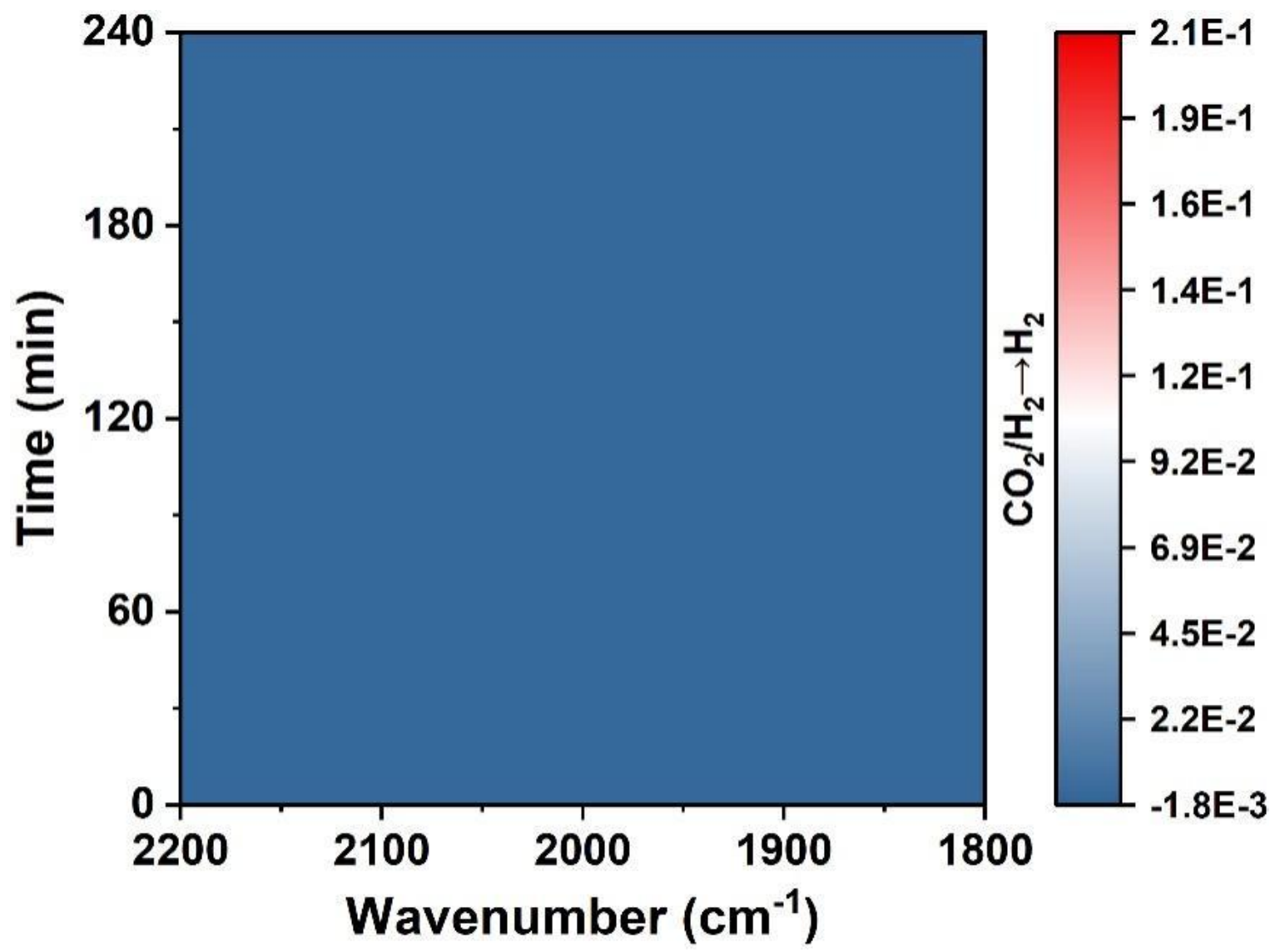

Figure S25. CO signal in CO_2 hydrogenation on PdCu@Y.

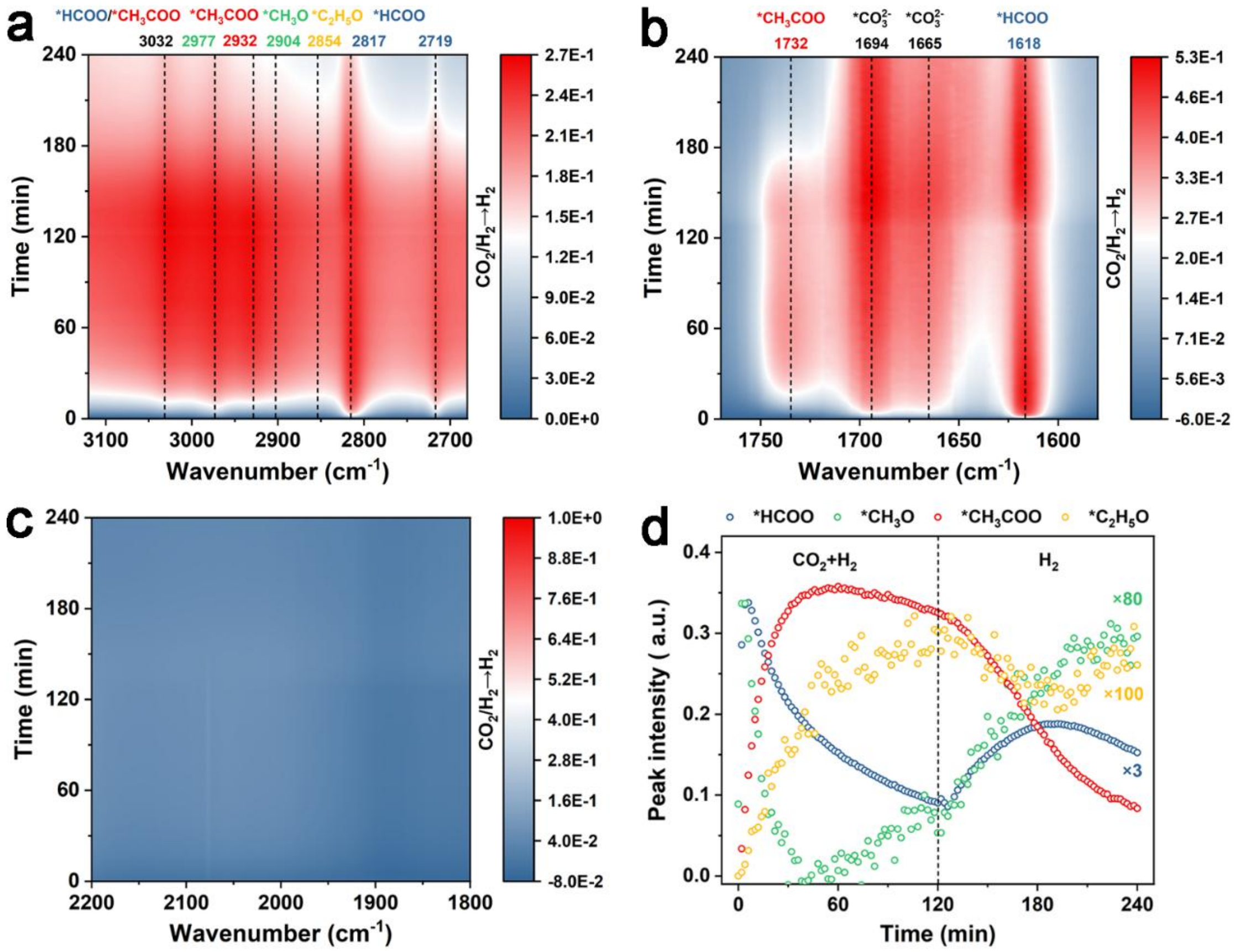

Figure S26. *In-situ* CO_2 hydrogenation DRIFTS on (a-c) PdCu@Y at 1 MPa and 200 ºC, (d) peak intensity changes versus reaction time.

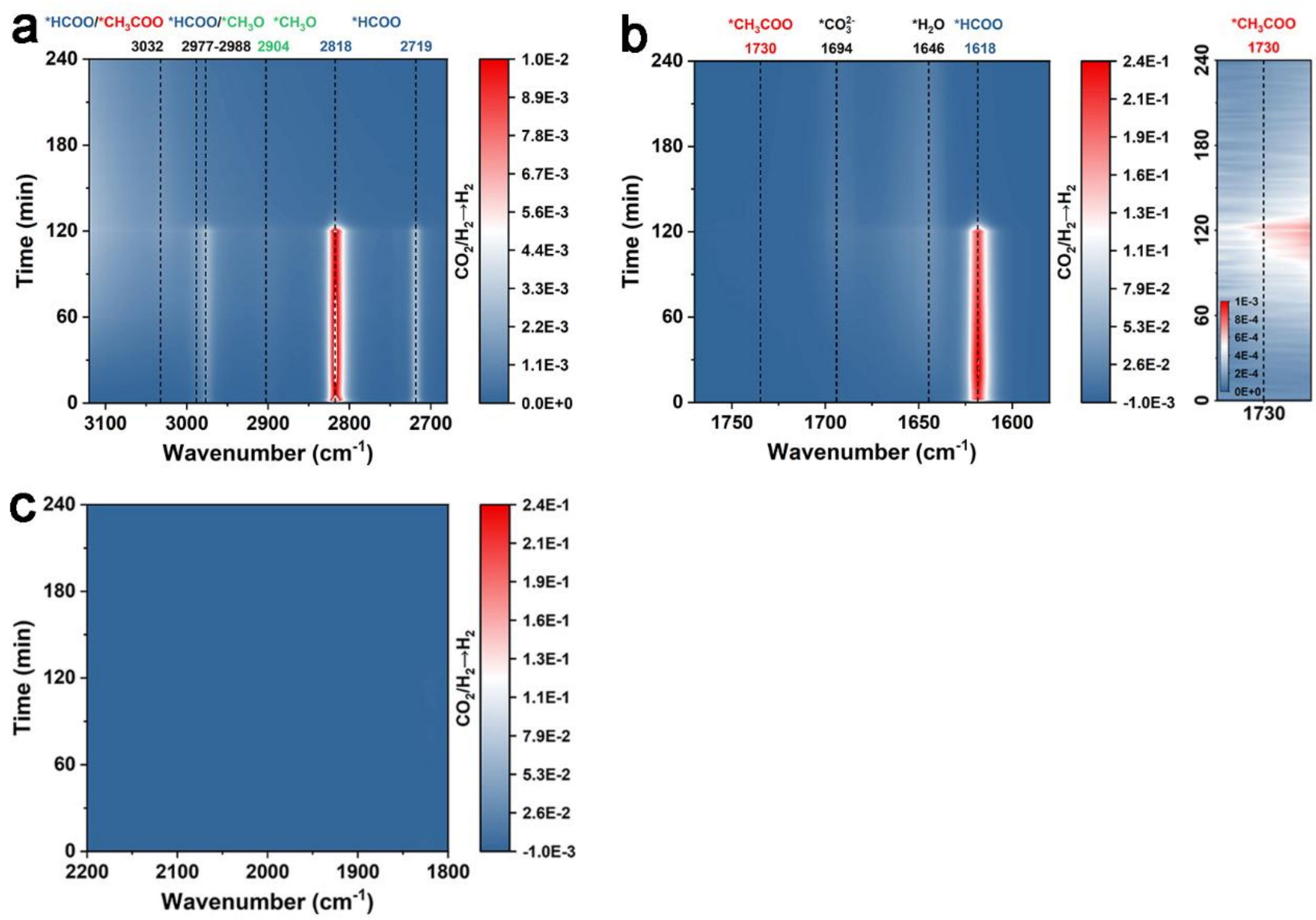

Figure S27. *In-situ* CO_2 hydrogenation DRIFTS on Pd@Y catalyst at 0.1 MPa and 200 ºC.

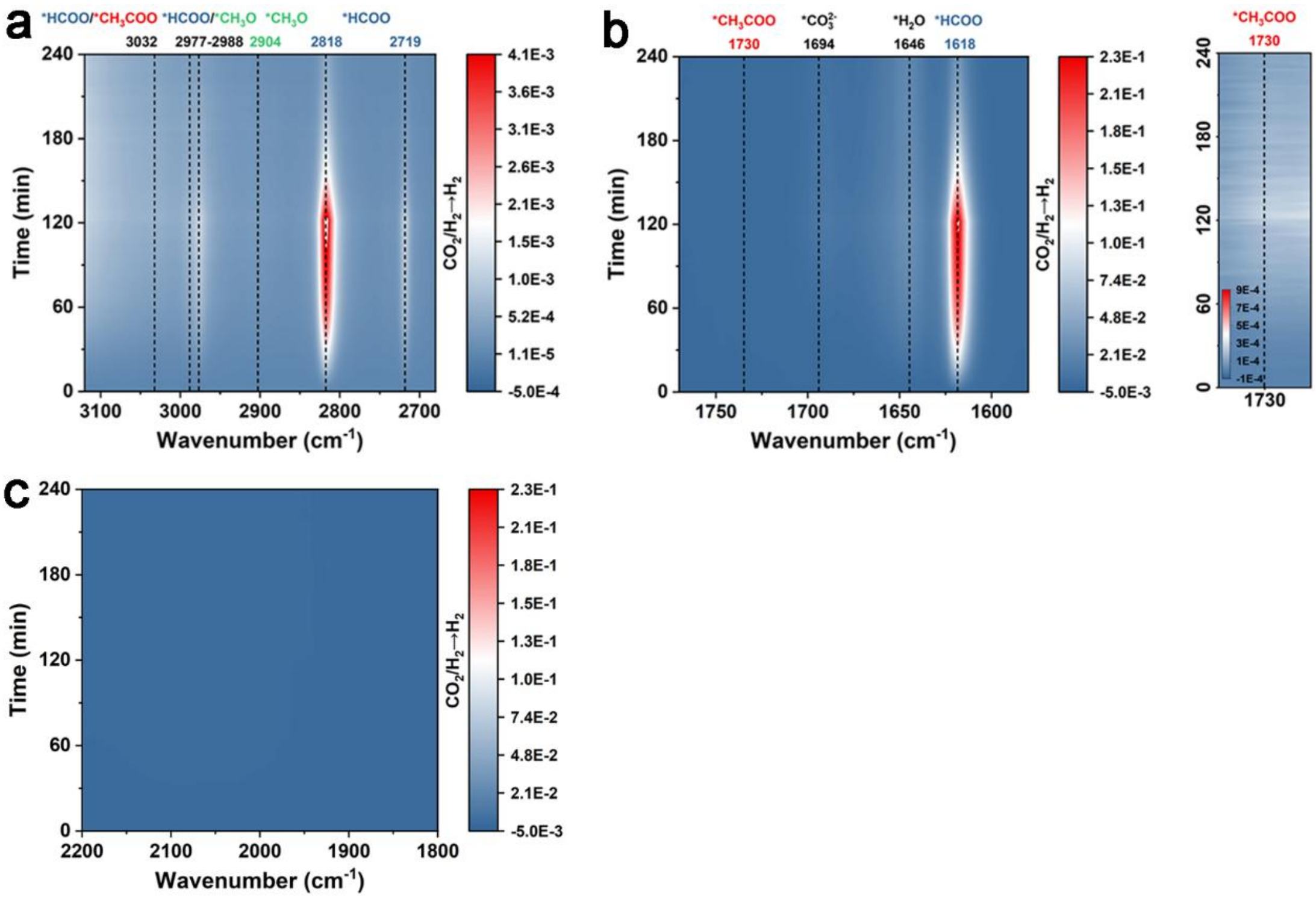

Figure S28. *In-situ* CO_2 hydrogenation DRIFTS on PdCu/Y catalyst at 0.1 MPa and 200 ºC.

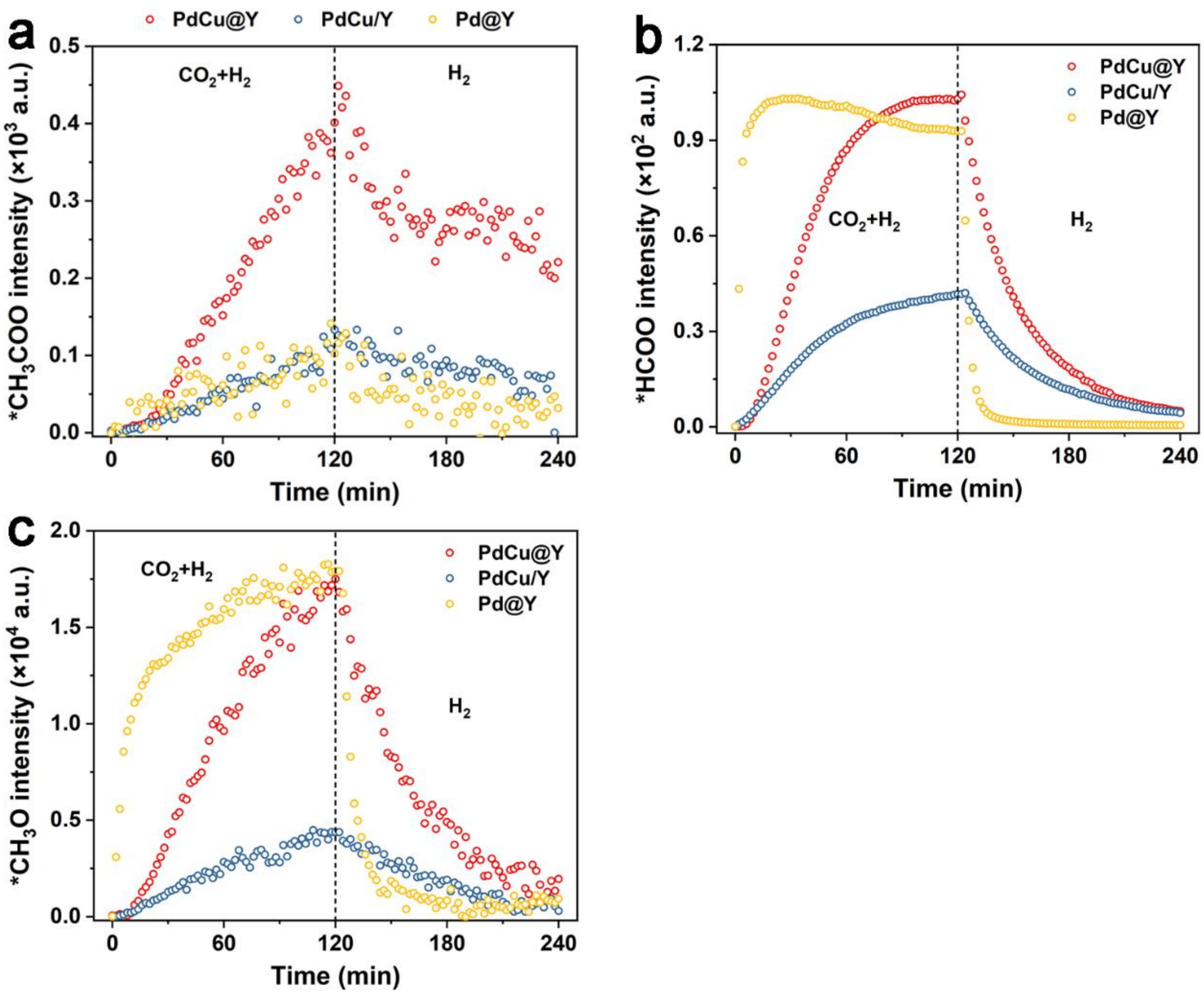

Figure S29. (a) *CH_3COO, (b) *HCOO and (c) *CH_3O peak intensity changes versus reaction time.

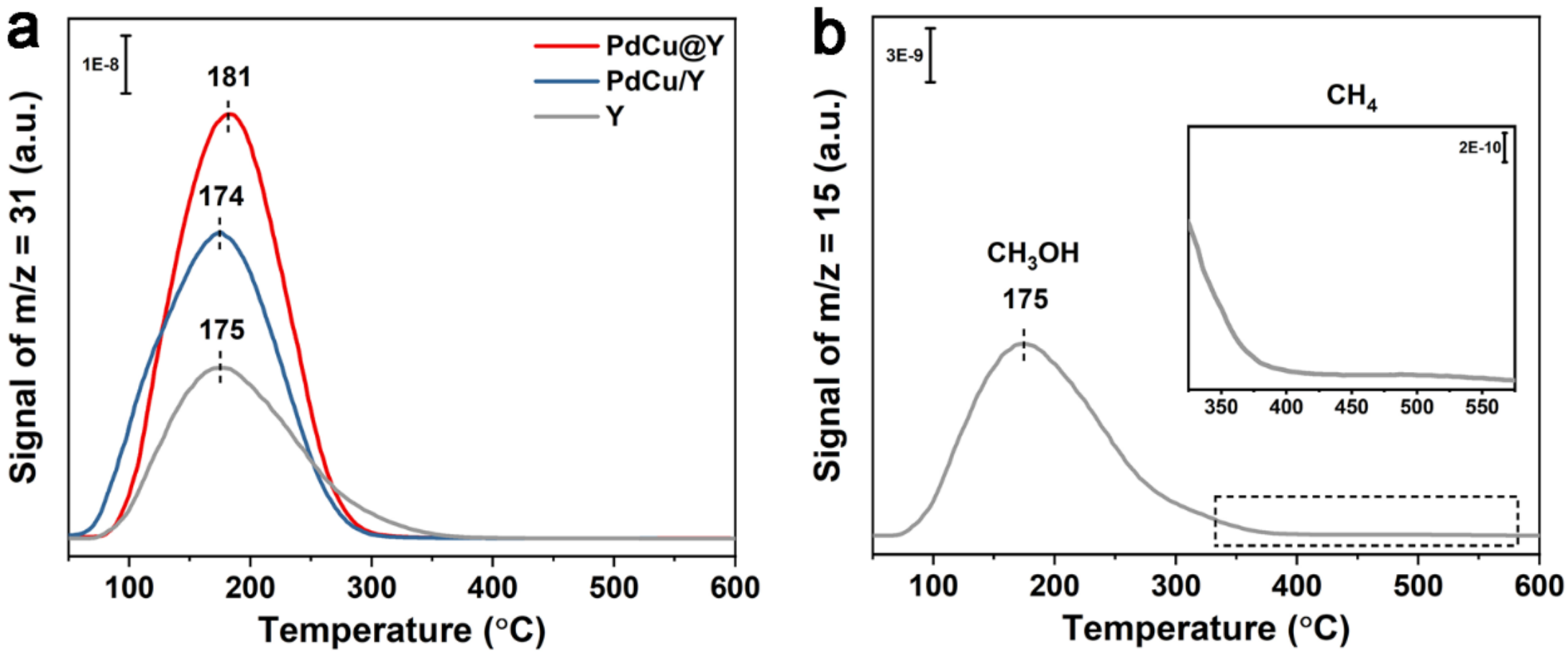

Figure S30. Signal of (a) m/z = 31 and (b) m/z = 15 in methanol temperature programmed surface reaction under H_2 atmosphere on PdCu@Y, PdCu/Y and Y.

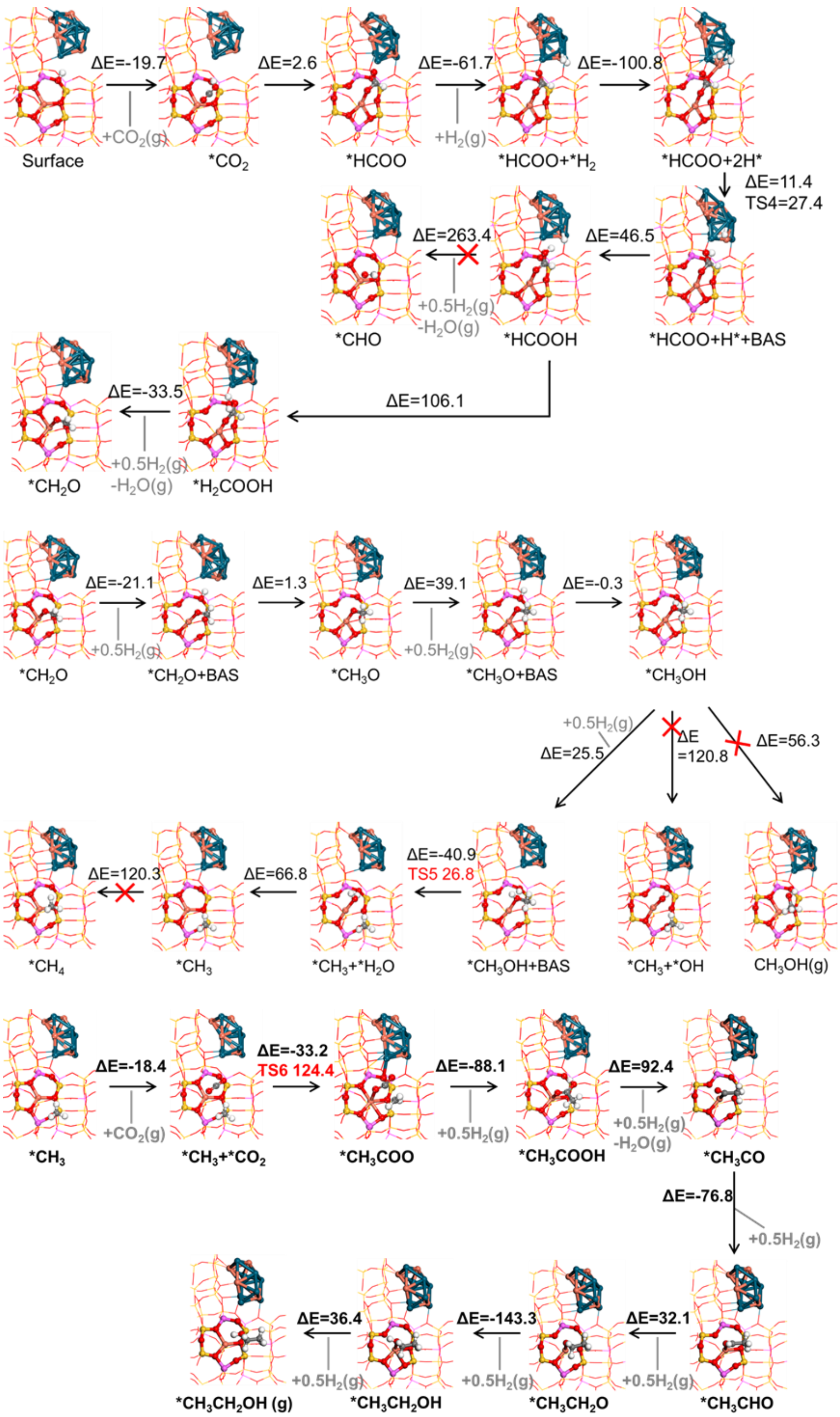

Figure S31. Structural models of the intermediates and the corresponding energy values involved in CO_2 hydrogenation to ethanol on PdCu-Y synergetic sites; the units of energies are in kJ mol^{-1}. Pd (blue), Cu (orange), Si (yellow), Al (pink), C (gray), O (red), H (white).

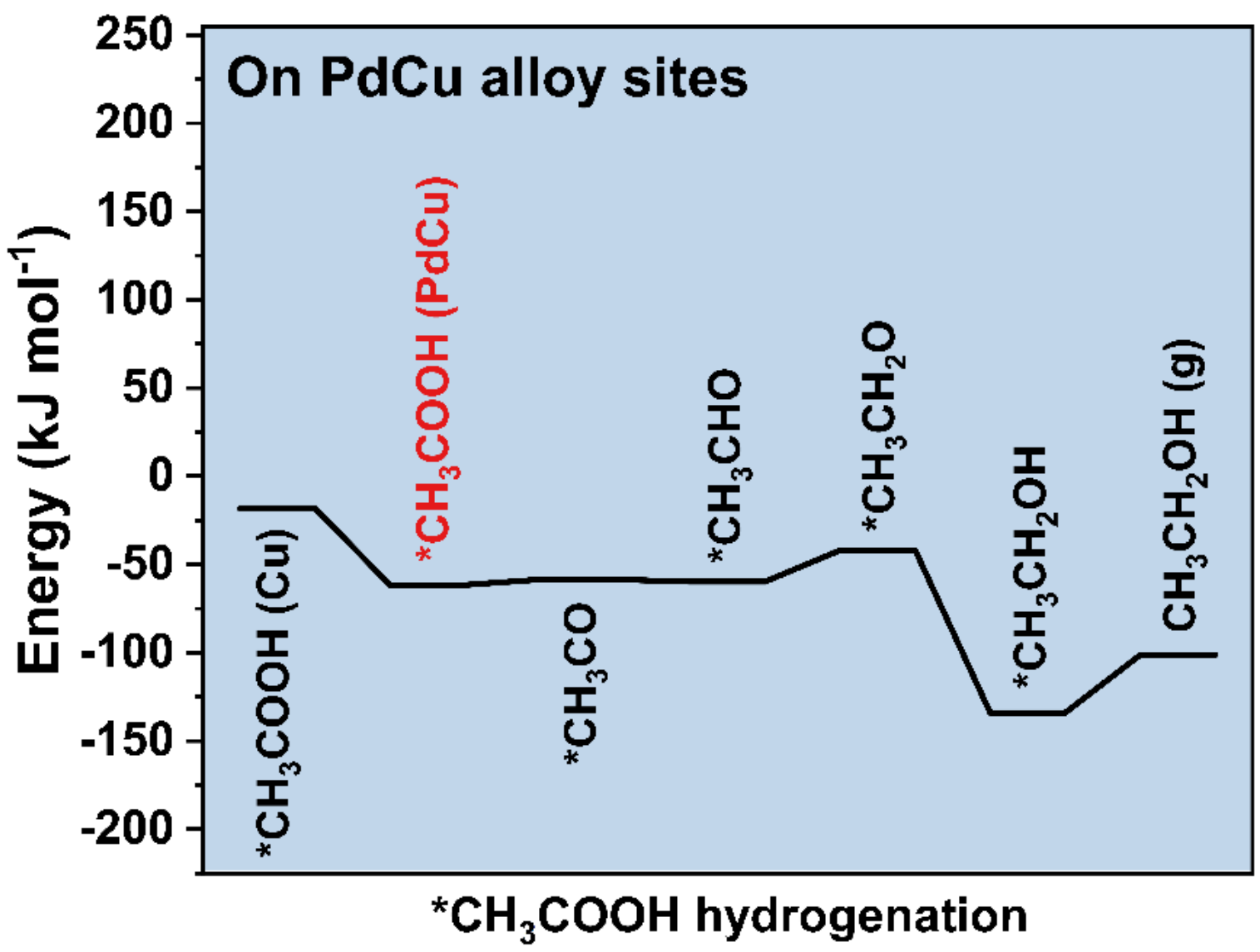

Figure S32. Reaction energy profile of $^*CH_3COOH$ hydrogenation to ethanol on PdCu alloy sites.

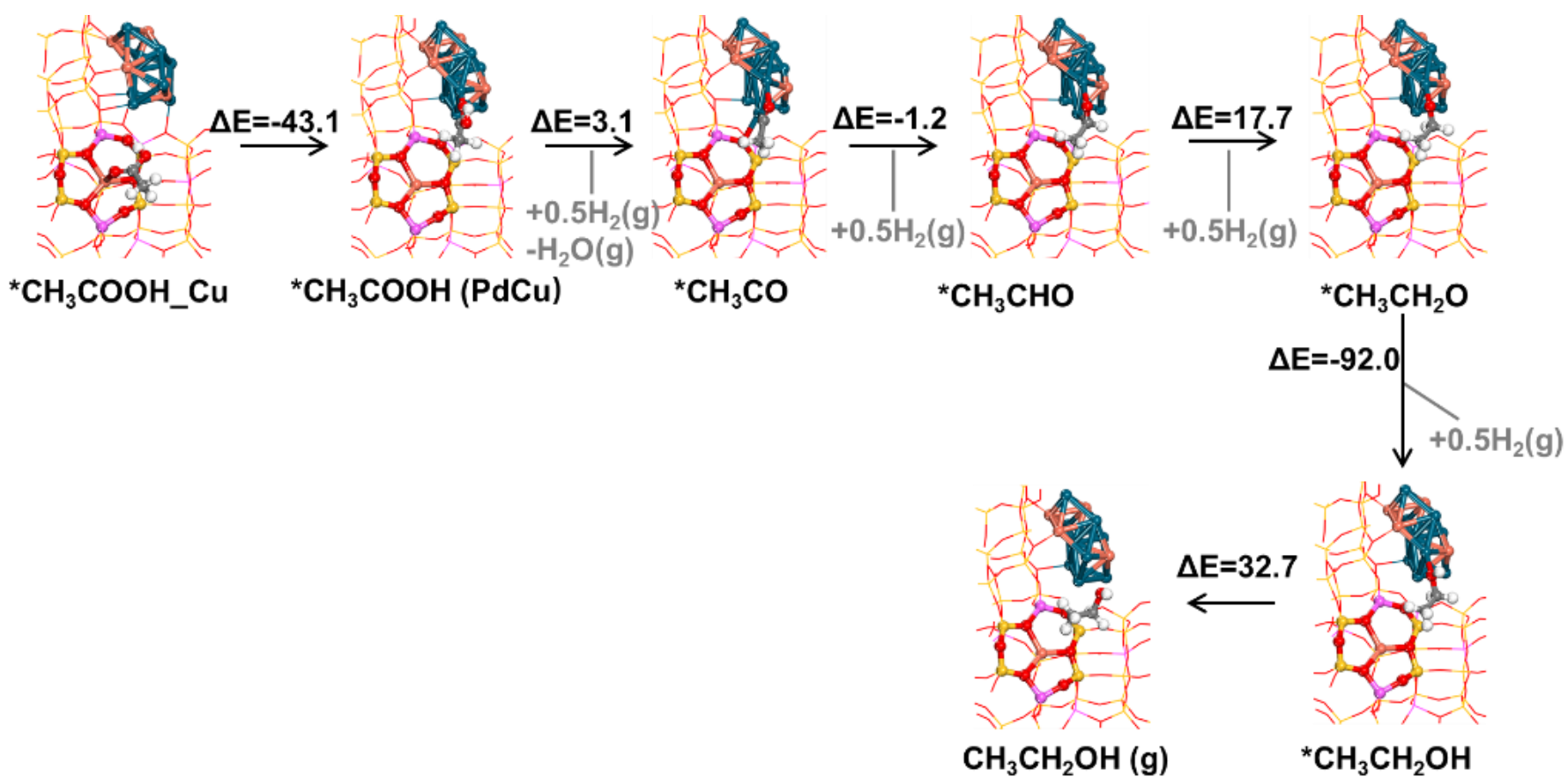

Figure S33. Structural models of the intermediates and the corresponding energy values involved in *CH_3COOH hydrogenation to ethanol on PdCu alloy sites; the units of energies are in kJ mol^{-1}. Pd (blue), Cu (orange), Si (yellow), Al (pink), C (gray), O (red), H (white).

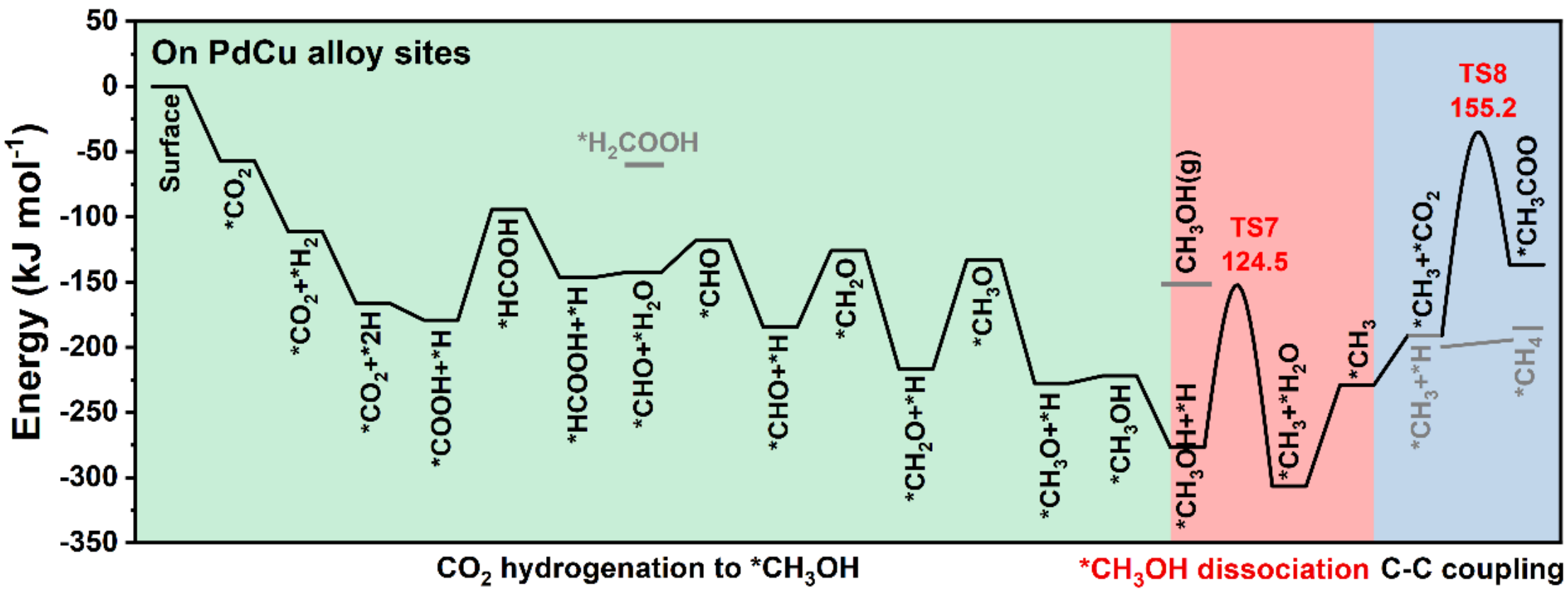

Figure S34. DFT-calculated reaction energy profiles of CO_2 hydrogenation on PdCu alloy sites.

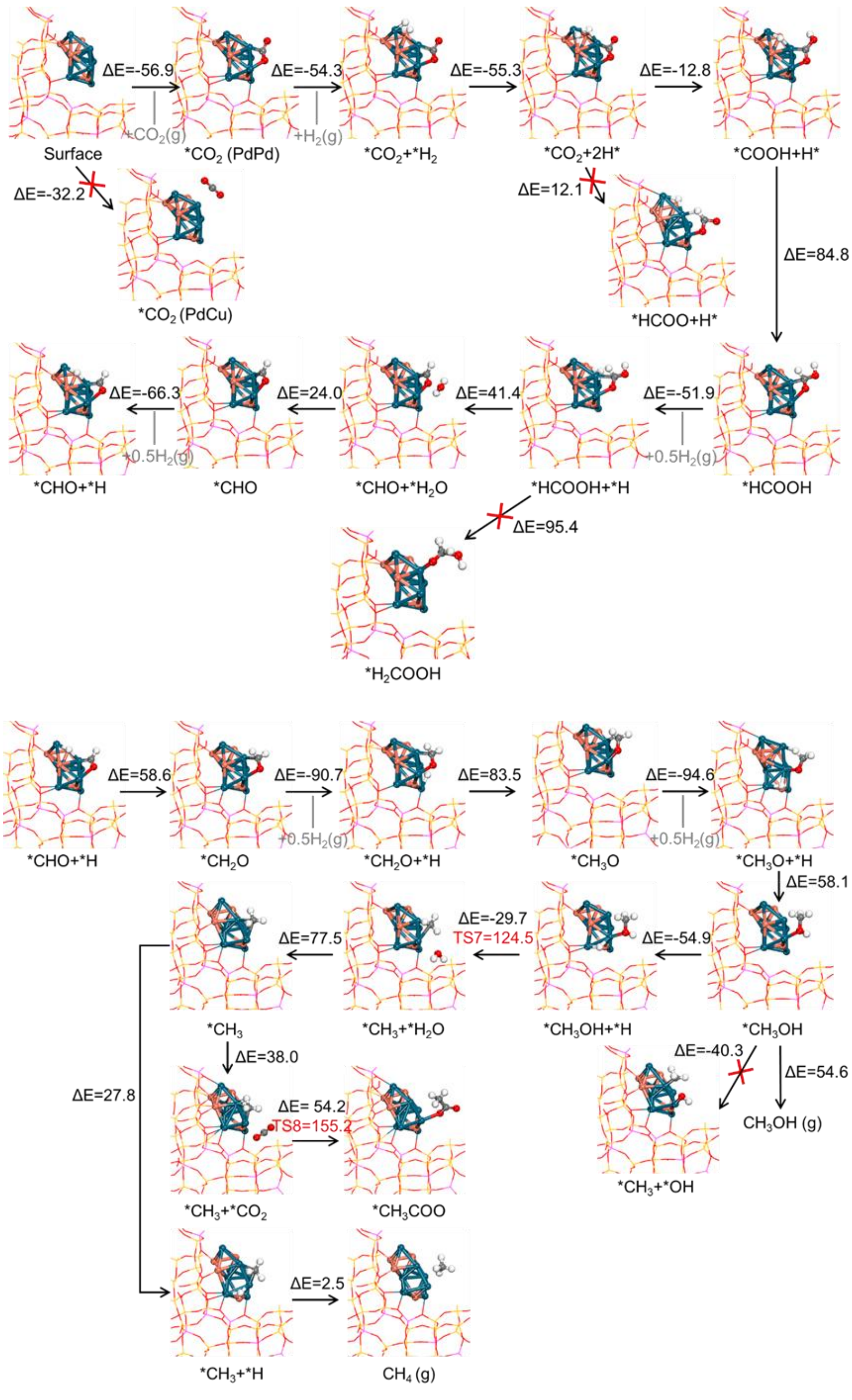

Figure S35. Structural models of the intermediates and the corresponding energy values involved in CO_2 hydrogenation on PdCu alloy sites; the units of energies are in kJ mol^{-1}. Pd (blue), Cu (orange), Si (yellow), Al (pink), C (gray), O (red), H (white).

References

1. Louwen, J. N., Simko, S., Stanciakova, K., Bulo, R. E., Weckhuysen, B. M., Vogt, E. T. C. Role of rare earth ions in the prevention of dealumination of zeolite Y for fluid cracking catalysts. *J. Phys. Chem. C* **124**, 4626-4636 (2020).
2. Chai, Y., Qin, B., Li, B., Dai, W., Wu, G., Guan, N., Li, L. Zeolite-encaged mononuclear copper centers catalyze CO_2 selective hydrogenation to methanol. *Nat. Sci. Rev.* **10**, nwad043 (2023).
3. Xu, D., Fan, H., Hou, G., Liu, K., Li, Y., Huang, S., Ding, M. Constructing stable Cu-Fe_5C_2 interfaces for efficient syngas conversion to higher alcohols. *Chem. Eng. J.* **499**, 155624 (2024).
4. Prins, R. Hydrogen spillover. Facts and fiction. *Chem. Rev.* **112**, 2714-2738 (2012).
5. Wang, Y., Wang, W., He, R., Li, M., Zhang, J., Cao, F., Liu, J., Lin, S., Gao, X., Yang, G., Wang, M., Xing, T., Liu, T., Liu, Q., Hu, H., Tsubaki, N., Wu, M. Carbon-based electron buffer layer on ZnO_x-Fe_5C_2-Fe_3O_4 boosts ethanol synthesis from CO_2 hydrogenation. *Angew. Chem. Int. Ed.* **62**, e202311786 (2023).
6. He, K., Li, Y., Li, Z., Chen, J., Tan, L., Qiu, T., Li, X., Ma, X., Dai, C. Enhanced ethanol synthesis via CO_2 hydrogenation using La-doped $CuFeO_x$ catalysts. *Chem. Eng. J.* **501**, 157608 (2024).
7. Du, P., Fakir, A. A. E., Zhao, S., Dostagir, N. H. M. D., Pan, H., Ting, K. W., Mine, S., Qian, Y., Shimizu, K. I. Ethanol synthesis via catalytic CO_2 hydrogenation over multi-elemental KFeCuZn/ZrO_2 catalyst. *Chem. Sci.* **15**, 15925 (2024).
8. Huang, J., Zhang, G., Wang, M., Zhu, J., Ding, F., Song, C., Guo, X. The synthesis of higher alcohols from CO_2 hydrogenation over Mn-Cu-K modified Fe_5C_2 and CuZnAlZr tandem catalysts. *Front. Energy Res.* **10**, 995800 (2023).
9. Ma, Y., Liu, Y., Huang, Z., Han, X., Ye, L., Qin, X., Xu, H., Kong, L., Li, J., Zhang, J., Pu, X., Liu, J. Ga modulation of C1 intermediates at Cu-FeO_x interfaces for efficient CO_2 hydrogenation to ethanol. *Sep. Purif. Technol.* **364**, 132583 (2025).
10. De Paula, L. D. N. R., Gomes, J. F., Assaf, J. M. Impact of the copper load on the performance of Cu/UiO-67 in CO_2 hydrogenation to ethanol at atmospheric pressure. *Catal. Today* **444**, 115014 (2025).
11. Ding, X., Fu, J., Lyu, Y., Ma, L., Xu, Y., Liu, X. Active sites of the cobalt catalysts controlled by surface silanols of silicalite-1 in CO_2 hydrogenation to ethanol. *Chem. Eng. J.* **494**, 152923 (2024).
12. Das, S., Tiwari, C. P., Yadav, G. D. Sustainable ethanol production via CO_2 hydrogenation with enhanced metal-support interfaces. *Ind. Eng. Chem. Res.* **64**, 5170-5183 (2025).
13. Lage, V. D., Valant, A. L., Bion, N., Toniolo, F. S. Tuning Co-Cu-Al catalysts and their reaction conditions on the CO_2 hydrogenation reaction to higher alcohols under mild conditions. *Chem. Eng. Sci.* **281**, 119208 (2023).
14. Zhang, S., An, K., Xin, J., Jiang, Y., Niu, M., Song, P., Wu, C., Wang, H., Liu, Y. Carbon modified active pairs of Co-Co_2C for CO_2 hydrogenation to alcohols. *Chem. Eng. J.* **486**, 150334 (2024).
15. Zhang, Y., Fan, G., Zheng, L., Li, F. Synergistic surface-interface catalysis in potassium-loaded Cu/CoO_x catalysts to boost ethanol production from CO_2

hydrogenation. *ACS Appl. Mater. Inter.* **17**, 13747-13761 (2025).
16. Zhang, J., Shao, Y., Zeng, F., Chen, H. Tuning the CO_2 hydrogenation activity and selectivity of TiO_2 nanorods supported Rh catalyst via secondary-metals addition. *AIChE J.* **70**, e18543 (2024).
17. Tran, C. C., Kaliaguine, S. Rhodium-doped iron oxides promoted by sodium for highly selective hydrogenation of CO_2 to ethanol and C_{2+} hydrocarbons. *Chem. Eng. J.* **496**, 153636 (2024).
18. Chen, Y., Zhou, D., Chang, Y., Xu, Y., Lin, H., Wu, L., Tang, Y., Dai, C., Li, X., Guo, L., Qiu, T., Tan, L. Rh-based bimetallic alloys: Unraveling the synergistic catalysis in CO_2 hydrogenation to ethanol. *ACS Catal.* **15**, 10068-10081 (2025).
19. Ji, S., Hong, F., Mao, D., Guo, Q., Yu, J. Enhanced ethanol synthesis from CO_2 hydrogenation over Fe and Na co-modified Rh/CeO_2 catalysts. *Chem. Eng. J.* **495**, 153633 (2024).
20. Chen, J., Zha, Y., Liu, B., Li, Y., Xu, Y., Liu, X. Rationally designed water enriched nano reactor for stable CO_2 hydrogenation with near 100% ethanol selectivity over diatomic palladium active sites. *ACS Catal.* **13**, 7110-7121 (2023).
21. Zhang, F., Zhou, W., Xiong, X., Wang, Y., Cheng, K., Kang, J., Zhang, Q., Wang, Y. Selective hydrogenation of CO_2 to ethanol over sodium-modified rhodium nanoparticles embedded in zeolite silicalite-1. *J. Phys. Chem. C* **125**, 24429-24439 (2021).
22. Caparros, F. J., Soler, L., Rossel, M. D., Angurell, I., Piccolo, L., Rossel, O., Llorca, J. Remarkable CO_2 hydrogenation to ethanol on Pd/Fe_3O_4 single-atom catalyst. *ChemCatChem* **10**, 2365-2369 (2018).
23. Irshad, M., Chun, H. J., Khan, M. K., Jo, H., Kim, S. K., Kim, J. Synthesis of n-butanol-rich C_{3+} alcohols by direct CO_2 hydrogenation over a stable Cu-Co tandem catalyst. *Appl. Catal. B: Environ.* **340**, 123201 (2024).
24. An, K., Zhang, S., Wang, H., Li, N., Zhang, Z., Liu, Y. Co^0- $Co^{\delta+}$ active pairs tailored by Ga-Al-O spinel for CO_2-to-ethanol synthesis. *Chem. Eng. J.* **43**, 134606 (2022).
25. Zhou, C., Aitbekova, A., Liccardo, G., Oh, J., Stone, M., Mcshane, E. J., Werghi, B., Nathan, S., Song, C., Ciston, J., Bustillo, K. C., Hoffman, A., Hong, J., Perez-Aguilar, J., Bare, S., Cargnello, M. Steam-assisted selective CO_2 hydrogenation to ethanol over Ru-In catalysts. *Angew. Chem. Int. Ed.* **63**, e202406761 (2024).
26. Kishida, M., Yamada, K., Nagata, H., Wakabayashi, K. CO_2 hydrogenation for C_{2+} alcohols synthesis over silica-supported Ir-Mo catalysts. *Chem. Lett.* **3**, 555-556 (1994).
27. Bando, K. K., Soga, K., Kunimori, K., Arakawa, H. Effect of Li additive on CO_2 hydrogenation reactivity of zeolite supported Rh catalysts. *Appl. Catal. A: Gen.* **175**, 67-81 (1998).
28. Kusama, H., Okabe, K., Sayama, K., Arakawa, H. CO_2 hydrogenation to ethanol over promoted Rh/SiO_2 catalysts. *Catal. Today* **28**, 261-266 (1996).
29. Kusama, H., Okabe, K., Sayama, K., Arakawa, H. Ethanol synthesis by catalytic hydrogenation of CO_2 over Rh-Fe/SiO_2 catalysts. *Energy* **22**, 343-348 (1997).
30. Gogate, M. R., Davis, R. J. Comparative study of CO and CO_2 hydrogenation over

supported Rh-Fe catalysts. *Catal. Commun.* **11**, 901-906 (2010).
31. Yang, C., Mu, R., Wang, G., Song, J., Tian, H., Zhao, Z., Gong, J. Hydroxyl-mediated ethanol selectivity of CO_2 hydrogenation. *Chem. Sci.* **10**, 3161-3167 (2019).
32. Wang, G., Luo, R., Yang, C., Song, J., Xiong, C., Tian, H., Zhao, Z., Mu, R., Gong, J. Active sites in CO_2 hydrogenation over confined VO_x-Rh catalysts. *Sci. Chi. Chem.* **62**, 1710-1719 (2019).
33. Ding, L., Shi, T., Gu, J., Cui, Y., Zhang, Z., Yang, C., Chen, T., Lin, M., Wang, P., Xue, N., Peng, L., Guo, X., Zhu, Y., Chen, Z., Ding, W. CO_2 hydrogenation to ethanol over Cu@Na-Beta. *Chem* **6**, 2673-2689 (2020).
34. Ye, X., Yang, C., Pan, X., Ma, J., Zhang, Y., Ren, Y., Liu, X., Li, L., Huang, Y. Highly selective hydrogenation of CO_2 to ethanol via designed bifunctional Ir_1-In_2O_3 single-atom catalyst. *J. Am. Chem. Soc.* **142**, 19001-19005 (2020).
35. Liu, Y., Liu, J., Li, G., Shi, X., Yin, J., Zheng, S., Yung, K. F., Yang, H., Lo, T. W. B. Exceptional CO_2 hydrogenation to ethanol via precise single-atom Ir deposition on functional P islands. *Angew. Chem. Int. Ed.* **64**, e202422744 (2025).
36. Gong, C., Wang, H., Zhang, J., Yang, C., Bu, X., Yang, H., Li, J., Gao, P. Construction of active Rh-TiO_x interfacial sites on $RhFeO_x$/P25 for highly efficient hydrogenation of CO_2 to ethanol. *ACS Catal.* **14**, 17582-17597 (2024).
37. Zheng, K., Li, Y., Liu, B., Jiang, F., Xu, Y., Liu, X. Ti-doped CeO_2 stabilized single-atom rhodium catalyst for selective and stable CO_2 hydrogenation to ethanol. *Angew. Chem. Int. Ed.* **61**, e202210991 (2022).
38. Zheng, K., Li, Y., Liu, B., Chen, J., Xu, Y., Li, Z., Liu, X. Phosphorus-substituted atomically dispersed Rh-N_3P_1 sites for efficient promotion in CO_2 hydrogenation towards ethanol production. *Appl. Catal. B: Environ. & Energy* **346**, 123730 (2024).
39. He, Z., Qiang, Q., Ma, J., Meng, Q., Zhou, H., Song, J., Liu, Z., Han, B. Water-enhanced synthesis of higher alcohols from CO_2 hydrogenation over a Pt/Co_3O_4 catalyst under milder conditions. *Angew. Chem. Int. Ed.* **55**, 737-741 (2016).
40. Wang, D., Bi, Q., Yin, G., Zhao, W., Huang, F., Xie, X., Jiang, M. Direct synthesis of ethanol via CO_2 hydrogenation using supported gold catalysts. *Chem. Commun.* **52**, 14226-14229 (2016).
41. Iltsiou, D., Mielby, J., Kegnaes, S. Direct conversion of CO_2 into alcohols using Cu-based zeolite catalysts. *ChemPlusChem* **89**, e202300313 (2024).
42. Wang, Z., Yang, C., Li, X., Song, X., Pei, C., Zhao, Z. J., Gong, J. The role of CO_2 dissociation in CO_2 hydrogenation to ethanol on CoCu/silica catalysts. *Nano Res.* **16**, 6128-6133 (2023).
43. Huang, Y., Guo, M., Wu, L., Ding, J., Zhong, Q. CO_2 hydrogenation to ethanol over Pt-Co_3O_4 catalysts: Effects of Pt promoter. *J. Environ. Sci.* **157**, 524-533 (2025).
44. Wang, L., Wang, L., Zhang, J., Liu, X., Wang, H., Zhang, W., Yang, Q., Ma, J., Dong, X., Yoo, S., Kim, J. G., Meng, X., Xiao, F. Selective hydrogenation of CO_2 to ethanol over cobalt catalysts. *Angew. Chem. Int. Ed.* **57**, 1-6 (2018).
45. Wang, L., He, S., Wang, L., Lei, Y., Meng, X., Xiao, F. Cobalt-nickel catalysts for selective hydrogenation of carbon dioxide into ethanol. *ACS Catal.* **9**, 11335-11340 (2019).

46. Bielanski, A., Datka, J. Amphiprotic properties of OH groups in synthetic NaHY zeolites. *J. Catal.* **32**, 183-189 (1974).
47. Kukulska-Zajac, E., Gora-Marek, K., Datka, J. IR and TPD studies of the reaction of acetic acid in zeolites NaHY. *Micropor. Mesopor. Mater.* **96**, 216-221 (2006).
